# Effects of different drying methods on the aroma and metabolic profiles of *Chrysanthemum morifolium* cv. Chuju

**DOI:** 10.1016/j.fochx.2026.104634

**Published:** 2026-10-21

**Authors:** Yu-fang Luo, Sam Al-Dalali, Su-ting Gao, Qi Shen, Yuan Fu, Yu-min Zhang, Wen Nie, Ye-ye Du

**Affiliations:** a School of Modern Agriculture and Food Engineering, Nutrition and Safety Research Laboratory of Anhui Cuisine, Chuzhou University, Chuzhou 239001, China; b Department of Food Science and Technology, Faculty of Agriculture and Food Science, Ibb University, Ibb 70270, Yemen; c Anhui Shunxin Shengyuan Biological Food Co., Ltd., Chuzhou 239064, China

**Keywords:** *Chrysanthemum morifolium* cv. Chuju, Drying methods, Sensory quantitative descriptive analysis, Electronic nose, GC-IMS, Untargeted metabolomics

## Abstract

*Chrysanthemum morifolium* cv. Chuju is a high-value edible flower with unique aroma characteristics but is susceptible to quality deterioration during drying. This study systematically evaluated the effects of four drying methods, including natural drying (ND), hot-air drying (HAD), microwave drying (MD), and vacuum freeze-drying (VFD), on the aroma profiles and metabolic characteristics of Chuju using sensory quantitative descriptive analysis (QDA), electronic nose (*E*-nose), gas chromatography-ion mobility spectrometry (GC–IMS), and untargeted metabolomics. A total of 90 volatile compounds and 2605 metabolites were identified. Drying treatments significantly altered volatile fingerprints and metabolic profiles. HAD exhibited higher signal intensities of several volatile compounds potentially associated with heat-induced transformations, whereas VFD showed relatively higher levels of aroma-related volatile compounds and thermolabile metabolites. Metabolite–volatile correlation analysis indicated that aroma differentiation among drying treatments was associated with amino acid metabolism, lipid oxidation-related pathways, phenylpropanoid metabolism, and flavonoid-related metabolism. These findings provide insights into the metabolic basis underlying drying-induced aroma variation and contribute to the optimization of Chuju drying strategies for quality preservation.

## 1 Introduction

*Chrysanthemum morifolium* cv. Chuju (Chuju), a genuine high-value edible flower native to Chuzhou, Anhui Province, is one of the four famous chrysanthemum cultivars in China with a centuries-old history of application. Chuju possesses distinctive aroma and functional properties, which are mainly associated with terpenoids, flavonoid glycosides, and dicaffeoylquinic acids. Terpenoid compounds, including *β*-ocimene, borneol, myrcene, and 1,8-cineole, have been reported to contribute to its characteristic floral and green-grass aroma, whereas phenolic compounds contribute to its antioxidant potential (Chen et al., 2021; Gao et al., 2025; Hao et al., 2022). Preservation of these quality-related compounds is therefore important for maintaining the value of dried Chuju products.

Fresh Chuju contains high moisture content, making drying essential for storage stability and product utilization. However, drying processes can influence compound stability, volatile profiles, and metabolic composition, thereby affecting aroma characteristics and functional quality (Benković-Lačić et al., 2023; He et al., 2026; Liu et al., 2024). Different drying technologies exhibit distinct advantages and limitations. Natural drying is time-consuming and highly influenced by environmental conditions (Zhang et al., 2025), whereas thermal drying methods such as hot-air drying and microwave drying may accelerate transformation or degradation of heat-sensitive compounds (Shi et al., 2026; Su et al., 2020). Vacuum freeze-drying generally improves compound retention but is limited by equipment requirements and energy consumption (Liu et al., 2022). Therefore, understanding the effects of different drying strategies on aroma and metabolic profiles is important for optimizing Chuju processing.

Previous studies on Chuju have mainly focused on individual quality attributes, such as volatile composition, phenolic compounds, flavonoids, and antioxidant activity, using conventional analytical approaches (Gao et al., 2025; Ling et al., 2025; Yu et al., 2025). Although the effects of drying on selected compounds have been investigated, the relationships among drying conditions, volatile variation, and metabolic changes remain insufficiently understood. Studies on other chrysanthemum cultivars, including Gongju and Hangbaiju, have suggested that multi-omics approaches can provide insights into quality changes induced by processing (Benković-Lačić et al., 2023; Zhou et al., 2023). However, whether similar metabolic alterations occur in Chuju, which possesses a distinct aroma-related chemical profile, remains unclear.

Therefore, this study aimed to elucidate the associations between drying methods, aroma characteristics, and metabolic changes in Chuju. Four drying strategies (ND, HAD, MD, and VFD) were compared by integrating sensory QDA, *E*-nose, GC–IMS, and untargeted metabolomics. This study explored the associations among drying conditions, volatile fingerprints, differential metabolites, and metabolic pathways, providing a comprehensive multi-omics perspective for understanding aroma differentiation during Chuju drying and revealing potential metabolic responses underlying quality variation induced by different drying strategies.

## 2 Materials and methods

### 2.1 Materials

Fresh *Chrysanthemum morifolium* cv. Chuju (cv. ‘Chuzhou Gongju’) flowers were harvested at the full-bloom stage in mid-November 2025 from a standardized cultivation farm located in Chuzhou, Anhui Province, China. Flowers with uniform size, color, and maturity were randomly selected, whereas samples with visible mechanical damage, disease symptoms, or insect infestation were excluded. The harvested flowers were transported to the laboratory under refrigerated conditions and processed within 24 h.

Methanol and acetonitrile (HPLC grade) were purchased from Merck (Darmstadt, Germany). Formic acid, acetic acid, and isopropanol (analytical grade) were obtained from Tianjin Kemiou Chemical Reagent Co., Ltd. (Tianjin, China). 4-Methyl-2-pentanol (CAS No. 108-11-2), used as the internal standard for GC–IMS analysis, was of analytical grade. All solvents used for UHPLC-MS/MS and GC–IMS analyses were of chromatographic grade.

### 2.2 Preparation of Chuju by different drying methods

Calyxes and pedicels were removed manually from fresh Chuju flowers. For each treatment, 100 g of fresh flowers was used as the initial sample. Four drying methods, including ND, HAD, MD, and VFD, were performed according to previously reported protocols with modifications (Bian et al., 2024; Yang et al., 2024; Yuan et al., 2015; Zhao et al., 2025). The same initial sample weight was used for all treatments, and each treatment included three biological replicates. The schematic procedures of the four drying methods are shown in Fig. S1. The drying parameters were selected based on previously reported conditions for edible chrysanthemum and similar plant materials, with modifications to achieve complete drying while avoiding excessive thermal damage. These conditions were not intended to represent optimized industrial parameters but rather to provide comparable processing conditions for evaluating aroma and metabolic differences among drying methods.

Samples were considered completely dried when the difference between two consecutive measurements was ≤0.002 g. Before weighing, samples were cooled to room temperature. For ND, the weighing interval was 24 h due to the long drying duration, whereas samples subjected to HAD, MD, and VFD were monitored according to drying progress until constant weight was reached.

For ND treatment, flowers were evenly distributed as a single layer on bamboo mats and dried naturally under shaded and well-ventilated conditions from mid-November to early December 2025. During drying, ambient temperature and relative humidity were monitored daily using a digital thermo-hygrometer. The average ambient temperature and relative humidity during the drying period were 5–15 °C and 30–50%, respectively. The drying process lasted approximately 20 days. For HAD treatment, flowers were spread as a 1-cm layer on stainless-steel trays and dried in a thermostatic drying oven (Model 10HA, Foshan Jinshen Electromechanical Equipment Co., Ltd., Foshan, China) at 70 °C until constant weight was achieved. The drying time was approximately 3 h. For MD treatment, flowers were placed in a microwave dryer (Model MKX-G1C1B, Qingdao Maikewei Microwave Innovation Technology Co., Ltd., Qingdao, China) and dried at 800 W with the temperature maintained at 60 °C until constant weight was reached. The total drying duration was approximately 60 min. For VFD treatment, flowers were pre-frozen at −20 °C for 7 h and subsequently transferred to a vacuum freeze dryer (Model FD-1 A-50, Shanghai Bilang Instrument Manufacturing Co., Ltd., Shanghai, China). Freeze-drying was performed at a cold trap temperature of −80 °C and vacuum pressure of 60 Pa for approximately 48 h until constant weight was obtained.

After drying, samples were ground, passed through a 40-mesh sieve, sealed in polyethylene bags, and stored at −20 °C until analysis.

### 2.3 Sensory quantitative descriptive analysis

This study adopted quantitative descriptive analysis (QDA) to characterize the aroma profile, rather than conducting consumer preference testing. Aroma contribution was evaluated based on relative intensity and multivariate correlation with volatile compounds; odor activity values (OAV) were not calculated due to the lack of concentration-threshold data. The experiment was performed with slight modifications based on the methodology reported by Zhao et al. (2025) and Li, Pu, et al. (2025). Thirty candidate evaluators were recruited from students majoring in Food Science, based on their previous experience in descriptive sensory analysis. All candidates underwent strict selection and training in accordance with ISO 8586:2012 (“Guidelines for the selection, training, and monitoring of trained expert sensory evaluators”) (ISO, 2012).

A first assessment was conducted for all 30 candidates after systematic training. Evaluators who scored below 80% in sensory identification and ranking tasks received supplementary training and then underwent a second assessment; those who failed the second evaluation were excluded from the panel. Finally, 10 qualified assessors were selected, who had received extensive training on the sensory characteristics of chrysanthemum products. Seven basic sensory attributes were determined through consensus discussion, including floral, sweet/honey, green-grass, fruity, woody, burnt/roasted, and stale/musty, and corresponding reference criteria were established (Table S1). The evaluators then received further training on these specific attributes and their corresponding reference standards.

Samples were randomly presented to evaluators using a balanced block design, and evaluations were carried out over three consecutive days, with each sample evaluated in duplicate. All sensory evaluations were performed in a standardized sensory laboratory maintained at 23 ± 2 °C and 50 ± 5% relative humidity, free of odor interference (Fig. S2). Participants were provided with tap water during the evaluation to ensure a comfortable and uninterrupted testing process.

All four sample groups were brewed with distilled water (90–100 °C) at a ratio of 1:50 (*w*/*v*) for 5 min. After brewing, the infusions were filtered through a food-grade 100-mesh tea-specific stainless-steel screen to remove suspended particles and ensure consistent sensory evaluation conditions. The filtered infusions were then transferred into 50-mL transparent, odorless disposable cups (30 mL per cup) and labeled with unique three-digit random numbers. Samples were freshly prepared and evaluated within 20 min, with a temperature maintained at 80 °C during testing. Before evaluating each sample, panelists rinsed their mouths with room-temperature water to avoid flavor carry-over.

Evaluators scored seven aroma attributes and overall aroma intensity using a 9-point intensity scale (0 = no detectable odor intensity; 3 = weak; 5 = moderate; 7 = strong; 9 = very strong), with higher scores representing stronger odor intensity. Each evaluator completed the scoring independently to eliminate bias. All participants signed the Sensory Evaluator Informed Consent Form approved by the Ethics Committee of Chuzhou University, confirming their voluntary participation, data confidentiality, and the right to withdraw from the study at any time.

### 2.4 Electronic nose analysis

*E*-nose analysis was performed using a PEN3 E-nose system (Airsense Analytics GmbH, Schwerin, Germany), with slight modifications to the method described by Wang, Dong, et al. (2025). Briefly, 1.0 g of dried, ground, and sieved Chuju sample was accurately weighed and transferred into a 40-mL headspace vial. The vial was hermetically sealed and equilibrated at room temperature before injection for E-nose detection. The optimized analytical parameters were as follows: sample interval = 1 s, auto-zero time = 10 s, measurement time = 80 s, purge time = 120 s, and carrier gas flow rate = 400 mL/min.

### 2.5 Tentative identification and relative profiling of volatile compounds

Volatile compounds were analyzed using a gas chromatography-ion mobility spectrometry (GC-IMS) system (FlavourSpec®, G.A.S., Dortmund, Germany). Briefly, 0.5 g of dried Chuju powder was accurately weighed and placed into a 20-mL headspace vial. Then, 0.5 mL of internal standard (4-methyl-2-pentanol, 2 mg/L) was added, and the vial was hermetically sealed. The sealed vial was incubated in a thermostatic water bath at 80 °C for 15 min with continuous magnetic stirring at 500 rpm. Adsorption was conducted at 80 °C for 30 min prior to thermal desorption at 250 °C for 5 min in the GC–IMS instrument.

An MXT-WAX capillary column (30 m × 0.53 mm ID × 1.0 μm df) was used for chromatographic separation, with high-purity nitrogen (purity ≥99.999%) as the carrier gas. The carrier gas flow rate program was set as follows: initial E1 flow rate = 150 mL/min and E2 flow rate = 2 mL/min at 0.00 min (start of data recording), maintained until 2.00 min; E2 flow rate was increased to 10 mL/min at 5.00 min (E1 flow rate remained at 150 mL/min); E2 flow rate was further increased to 100 mL/min at 25.00 min (E1 flow rate remained at 150 mL/min); data recording was terminated at 30.00 min. The IMS parameters were set as follows: drift tube temperature = 60 °C, positive ionization mode, drift tube length = 98 mm, linear voltage = 500 V/cm, and tritium (^3^H) as the radioactive ion source.

Data analysis was performed using VOCal software (G.A.S., Germany). The Reporter, Gallery Plot, and Dynamic Principal Component Analysis (PCA) plugins were used to visualize volatile organic compounds (VOCs) distributions and evaluate sample similarities. Volatile features were tentatively annotated based on the comparison of retention indices (RI), drift times, and migration characteristics with the NIST and VOCal databases. Since authentic standards were not individually analyzed for all detected compounds, these annotations should be considered putative identifications rather than definitive structural confirmation. The internal standard, 4-methyl-2-pentanol, was added to monitor analytical repeatability and signal variation among measurements. However, it was not used for absolute quantification because compound-specific calibration curves and response factors were unavailable. The relative abundance of each volatile feature was represented by its GC–IMS peak volume. These peak volumes were used for comparative profiling among different drying treatments rather than absolute quantification of individual compounds. Because the objective of this study was to characterize treatment-associated differences in volatile fingerprints, GC–IMS peak volumes were interpreted as relative semi-quantitative indicators. Absolute concentrations were not calculated because compound-specific calibration curves, recovery experiments, and matrix-effect corrections were not established for individual volatile compounds. zPCA further evaluated sample classification.

OAV were not calculated in this study. Although OAV analysis is commonly used to estimate the contribution of volatile compounds to aroma perception, reliable odor threshold values are unavailable for many VOCs identified by GC–IMS, particularly terpenoid derivatives, ester compounds, and other characteristic aroma components of Chuju. Furthermore, reported odor thresholds are generally obtained from different matrices (e.g., air, water, or model food systems) and may not accurately represent the aroma contribution within the Chuju matrix. Therefore, aroma characteristics were interpreted by integrating QDA, *E*-nose analysis, GC–IMS fingerprinting, and metabolomic results.

Although GC–IMS provides highly sensitive fingerprinting of volatile profiles in complex matrices, compound annotation relies on database matching and may be influenced by matrix effects. Therefore, the identified volatile compounds were interpreted as tentative annotations, and future studies using authentic standards and quantitative calibration are required for absolute confirmation.

### 2.6 Untargeted metabolomics analysis

A total of 12 independently prepared Chuju samples were analyzed, including four drying treatments (HAD, VFD, MD, and ND) with three biological replicates per group. All extraction procedures and LC-MS/MS measurements were performed under standardized conditions to ensure analytical consistency. For each sample, 25 mg of dried Chuju powder was accurately weighed and transferred into a centrifuge tube. Then, 1 mL of pre-chilled extraction solution (−40 °C), consisting of 40% methanol, 40% acetonitrile, and 20% water (*v*/v/v), was added. The mixture was homogenized at 35 Hz (6000 rpm) for 4 min, followed by sonication in an ice-water bath for 5 min. This homogenization-sonication cycle was repeated three times, and the mixture was then incubated at −40 °C for 1 h. A 300 μL aliquot was transferred to a 96-well filter plate connected to a collection plate, and the plate was placed in a positive-pressure chamber. The chamber was slowly pressurized to 6 psi and held for 3 min to collect the supernatant for ultra-high-performance liquid chromatography-tandem mass spectrometry (UHPLC-MS/MS) analysis. Equal volumes of all samples were mixed to prepare three quality control (QC) samples. QC samples were prepared by pooling equal volumes of all samples and were injected periodically throughout the analytical sequence to monitor instrument stability and analytical reproducibility. The reliability of the dataset was evaluated based on QC sample clustering in PCA, correlation coefficients among QC injections (>0.99), and relative standard deviation (RSD) values of internal standards (≤15%).

UHPLC-MS/MS analysis was carried out using a Vanquish ultra-high-performance liquid chromatograph coupled with a mass spectrometer (Thermo Fisher Scientific, Waltham, MA, USA), equipped with a Phenomenex Kinetex C18 column (2.1 mm × 50 mm, 2.6 μm). The mobile phase consisted of phase A (water containing 0.01% acetic acid, *v*/v) and phase B (isopropanol: acetonitrile = 1:1, *v*/v). The sample tray temperature was maintained at 4 °C, and the injection volume was 2 μL. The MS parameters were set as follows: sheath gas flow rate = 50 arb, auxiliary gas flow rate = 15 arb, capillary temperature = 320 °C, full MS resolution = 60,000, MS/MS resolution = 15,000, collision energies = 20/30/40 SNCE, and simultaneous scanning in positive and negative ion modes with spray voltages of 3.8 kV and − 3.4 kV, respectively.

Raw data were converted into mzXML format using ProteoWizard software (Version 3.0.24054). Metabolite annotation was conducted using the bioinformatics workflow provided by the metabolomics service provider, with reference to the in-house BT-Plant database (V1.1, plant-specific), Kyoto Encyclopedia of Genes and Genomes (KEGG, *Arabidopsis thaliana*), Human Metabolome Database (HMDB), and PubChem. Metabolite annotation confidence was evaluated according to the Metabolomics Standards Initiative (MSI) guidelines. Level 1 metabolites were identified by matching retention time (RT), precursor ion (MS1), and fragment ion spectra (MS2) with authentic reference standards. Level 2 metabolites were putatively annotated based on MS1 and MS2 spectral matching against public databases. Level 3 metabolites were assigned based on matching MS1 and MS2 information with putative molecular candidates without confirmation by authentic standards. Accordingly, annotated metabolites (Levels 1–3) were included in the subsequent analyses, whereas unidentified features (Level 4) were excluded from downstream biological interpretation.

Because untargeted metabolomics provides relative metabolic profiling rather than absolute quantification, differential metabolites were interpreted based on treatment-associated changes rather than confirmed quantitative concentrations. Further validation using authentic standards and targeted quantitative approaches is required for absolute confirmation of individual metabolites.

### 2.7 Statistical analysis of data

All experiments were performed in triplicate, and data are expressed as mean ± standard deviation (SD). Sensory evaluation, *E*-nose, and GC-IMS data were analyzed using SPSS 26.0 software (IBM Corp., Armonk, NY, USA). Differences among groups were analyzed by one-way analysis of variance (ANOVA) followed by Duncan's multiple range test. Differential metabolites were screened using VIP > 1, *P* < 0.05, and FC ≥ 2 or FC ≤ 0.5 as the criteria. Here, FC ≥ 2 and FC ≤ 0.5 represented at least a two-fold increase and a decrease to 50% or less, respectively.

MetaboAnalyst 6.0 (https://www.metaboanalyst.ca/) was used for KEGG pathway enrichment analysis and K-means clustering. The optimal cluster number was evaluated using both the Elbow Method and the Silhouette Coefficient (Fig. S3). The elbow plot indicated an inflection around k = 4, whereas the highest average silhouette coefficient was obtained at k = 3. Considering both clustering performance and biological interpretability, k = 3 was selected for the subsequent K-means clustering analysis.

To improve data reliability, semi-quantitative analysis of volatile compounds was performed using 4-methyl-2-pentanol as an internal standard. The relative content of volatile compounds was estimated based on the ratio between the peak volume of each identified compound and that of the internal standard. Since GC–IMS provides signal responses rather than absolute concentrations, the calculated values were considered relative semi-quantitative indicators and were used for comparative analysis among different drying treatments. For metabolomics, representative differential metabolites were compared based on normalized relative signal intensities. Absolute quantification was not conducted; all results are presented as relative abundance. This study provides comparative profiling rather than absolute quantitative determination. In accordance with Section 2.6, raw metabolomics data were converted into mzXML format using ProteoWizard (Version 3.0.24054) and subsequently processed using the standard bioinformatics pipeline provided by the metabolomics service provider. Data preprocessing included missing value imputation, outlier removal, total ion current (TIC) normalization, logarithmic transformation, and UV normalization. Raw data preprocessing and metabolite annotation were conducted according to the standardized workflow of the metabolomics service provider. In contrast, downstream statistical analyses, including KEGG pathway enrichment and K-means clustering, were performed using MetaboAnalyst 6.0.

## 3 Results and discussion

### 3.1 Effect of different drying methods on the aroma of Chuju

Visual appearances of Chuju samples treated with four different drying methods are shown in Fig. 1A, and sensory evaluation results of these dried samples are summarized in Fig. 1B. Distinct differences in aroma profiles were observed among the drying treatments. VFD samples exhibited relatively higher floral and green-grass aroma intensities, together with a relatively pronounced fruity note, indicating better retention of fresh-like aroma characteristics. This pattern may be related to the relatively mild drying environment of VFD, in which low temperature and reduced oxygen exposure may limit the thermal degradation and oxidation of aroma-related compounds.

**Fig. 1:**
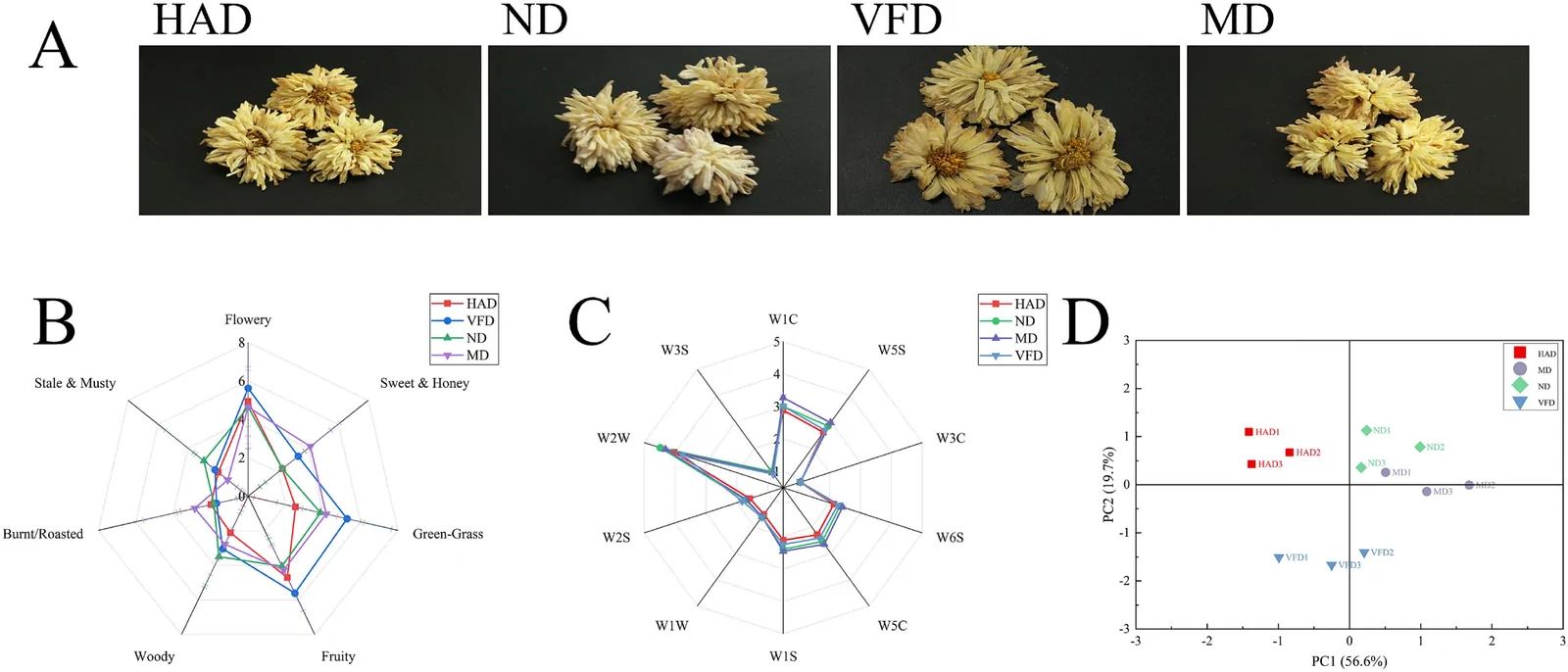
Effects of different drying methods on sensory characteristics and electronic nose (*E*-nose) profiles of Chuju. (A) Visual appearance of Chuju samples prepared by four drying methods: hot-air drying (HAD), natural drying (ND), vacuum freeze-drying (VFD), and microwave drying (MD). (B) Sensory aroma profile radar map of Chuju samples. (C) Response intensity of E-nose sensors to volatile components in Chuju. (D) Principal component analysis (PCA) score plot based on E-nose data. Data are presented as mean ± SD (*n* = 3) (*P* < 0.05).

Similar preservation effects have been reported in other chrysanthemum cultivars. For example, vacuum freeze-drying effectively retained phenolic compounds and volatile precursors in Gongju and other chrysanthemum varieties compared with conventional thermal drying (Fan et al., 2025; Wang et al., 2019). However, cultivar-specific differences should also be considered. Compared with Gongju and Hangbaiju, which are often characterized by abundant flavonoid-related functional components, Chuju exhibits a distinctive aroma profile with relatively rich terpenoid-derived volatiles (Chang et al., 2021; Li, Xu, et al., 2025; Zhang et al., 2025). Therefore, maintaining volatile precursors may be particularly important for Chuju aroma quality.

HAD samples showed a relatively pronounced sweet and honey-like aroma, whereas their green-grass aroma intensity was comparatively low. The enhanced sweet and honey-like perception may be associated with thermal-induced transformation of endogenous precursors. Previous studies on Gongju and Jinsihuangju have shown that hot-air drying has been reported to be associated with increased abundance of aldehydes, ketones, and other aroma-related compounds through amino acid degradation and carbohydrate-related reactions (Sun et al., 2026; Wang et al., 2018). Consistently, several aldehyde- and ketone-related volatile compounds, including 2,3-pentanedione and 3-methylbutanal, showed relatively higher signal intensities in HAD samples (Table 1). These compounds may contribute to the enhanced sweet and roasted aroma perception (Jia et al., 2023). However, prolonged exposure to high temperature may also accelerate the loss of thermolabile floral compounds, resulting in weaker fresh aroma characteristics compared with VFD. MD samples exhibited a relatively balanced aroma profile, with moderate intensities across several aroma attributes and a comparatively pronounced sweet and honey-like note. Compared with HAD, microwave treatment provides rapid volumetric heating and shorter dehydration time, which may reduce prolonged thermal exposure. Similar observations have been reported for Gongju, where microwave-assisted drying improved drying efficiency while maintaining relatively favorable quality characteristics (Wang et al., 2018). However, the specific influence of microwave treatment on Chuju aroma requires further investigation under different power and temperature conditions. ND samples showed relatively pronounced woody and stale/musty characteristics, whereas their overall fresh-like aroma characteristics were less prominent. This phenomenon may be related to the prolonged drying duration and uncontrolled environmental exposure. Previous studies on chrysanthemum products have suggested that extended natural drying may be associated with oxidative changes in phenolic compounds and lipid-derived aroma precursors (Fan et al., 2025). Nevertheless, because microbial activity and environmental microorganisms were not monitored in the present study, their potential contribution during long-term natural drying requires further investigation.

**Table 1:** Relative contents of 90 volatile compounds in Chuju under four drying methods (mean ± SD, *n* = 3).

No.	Compound	CAS	RI	Rt [sec]	HAD (a.u.)	MD (a.u.)	VFD (a.u.)	ND (a.u.)
Alcohols								
1	1-Terpinen-4-ol	562-74-3	1615.8	1685.671	1288.10 ± 97.51 ^c^	1418.01 ± 134.12^c^	3201.15 ± 300.00 ^a^	1869.44 ± 214.84^b^
10	1-Octen-3-ol	3391-86-4	1469.9	1061.281	682.57 ± 46.28^b^	446.57 ± 52.82 ^c^	3206.61 ± 403.99 ^a^	1736.83 ± 147.51 ^b^
19	Linalool	78-70-6	1393.6	833.292	172.88 ± 14.12 ^b^	157.02 ± 9.16 ^b^	311.73 ± 12.63 ^a^	200.91 ± 10.36 ^b^
20	(Z)-3-Hexen-1-ol	928-96-1	1380.5	799.448	618.33 ± 32.48 ^c^	662.17 ± 45.94 ^c^	1174.43 ± 107.62 ^a^	990.05 ± 28.68 ^b^
21	1-Hexanol-M	111-27-3	1363	756.058	529.91 ± 56.23^c^	468.33 ± 66.38 ^c^	1953.92 ± 23.66 ^a^	1641.80 ± 211.73 ^b^
22	1-Hexanol-D	111-27-3	1364	758.661	113.83 ± 15.21 ^c^	89.03 ± 9.48 ^c^	913.22 ± 60.06 ^a^	661.29 ± 165.71 ^b^
31	2-Butanone3-hydroxy	513-86-0	1293.7	607.061	603.83 ± 32.47 ^a^	519.82 ± 12.94 ^a^	335.05 ± 29.02 ^b^	643.78 ± 17.41 ^a^
35	1-Pentanol-M	71-41-0	1258.1	556.274	537.86 ± 14.72 ^b^	546.69 ± 12.93 ^b^	812.63 ± 9.24 ^a^	781.92 ± 9.33 ^a^
36	1-Pentanol-D	71-41-0	1256.6	554.113	195.23 ± 11.13 ^b^	189.47 ± 8.55 ^b^	821.16 ± 21.67 ^a^	654.50 ± 43.58 ^a^
49	(Z)-3-Hexen-1-ol	928-96-1	1190.7	471.451	105.50 ± 4.16 ^b^	233.51 ± 5.20 ^a^	125.92 ± 5.74 ^b^	139.86 ± 2.85 ^b^
51	1-Butanol-M	71-36-3	1143.5	417.963	268.57 ± 12.44 ^a^	228.49 ± 5.13 ^b^	297.30 ± 5.49 ^a^	269.96 ± 10.44 ^a^
52	1-Butanol-D	71-36-3	1143	417.423	75.90 ± 6.17 ^c^	45.15 ± 1.07 ^d^	189.80 ± 13.81 ^a^	128.15 ± 8.42 ^b^
58	1-Propanol,2-methyl-M	78-83-1	1094.5	368.434	141.91 ± 2.94 ^a^	118.84 ± 11.79 ^b^	129.15 ± 5.26a ^b^	116.75 ± 17.55 ^b^
59	1-Propanol,2-methyl-D	78-83-1	1093.4	367.412	102.86 ± 10.38 ^b^	67.27 ± 3.48 ^c^	296.35 ± 37.36 ^a^	286.70 ± 83.71 ^a^
68	3-Pentanol	584-02-1	988.6	291.872	456.27 ± 18.26 ^b^	411.80 ± 3.83 ^b^	559.72 ± 7.81 ^a^	589.50 ± 11.10 ^a^
81	2-Pentanol	6032-29-7	1128.8	402.443	405.47 ± 31.81 ^c^	697.11 ± 9.76 ^b^	925.87 ± 14.65 ^a^	662.31 ± 44.42 ^b^
88	2-Methyl-1-butanol	137-32-6	1211.8	496.464	146.29 ± 4.72 ^b^	120.64 ± 14.47 ^c^	201.40 ± 5.72 ^a^	201.92 ± 5.43 ^a^
89	1-Butanol,3-methyl-M	123-51-3	1211.4	495.997	113.83 ± 5.67 ^c^	83.79 ± 4.35d	220.83 ± 4.86 ^a^	147.31 ± 6.55 ^b^
90	1-Butanol,3-methyl-D	123-51-3	1210.7	495.064	232.49 ± 10.12 ^c^	239.34 ± 4.58 ^c^	577.61 ± 51.05 ^a^	294.42 ± 41.69 ^b^

Terpenes								
11	Furan linalool oxide	60047-17-8	1447.5	988.71	356.04 ± 36.17 ^b^	243.47 ± 10.54 ^c^	443.92 ± 37.77 ^a^	677.44 ± 15.00 ^a^
25	Sabinene	3387-41-5	1334.2	690.105	527.87 ± 46.07 ^a^	533.27 ± 25.26 ^a^	421.70 ± 13.93 ^b^	513.66 ± 34.91 ^a^
30	β-Myrcene	123-35-3	1313.1	645.42	371.83 ± 23.30 ^d^	765.51 ± 17.92 ^c^	1105.82 ± 12.23 ^a^	515.64 ± 28.92 ^d^
33	α-Terpinolene	586-62-9	1285.7	595.174	813.34 ± 59.46 ^c^	790.95 ± 24.30 ^c^	1891.64 ± 46.25 ^a^	1198.12 ± 20.70 ^b^
38	γ-Terpinene	99-85-4	1243.6	536.825	3108.46 ± 154.84 ^c^	4085.78 ± 45.82 ^b^	4555.33 ± 78.85 ^a^	3354.86 ± 97.33 ^c^
39	β-Ocimene	13877-91-3	1232.9	522.777	633.75 ± 71.98 ^c^	735.61 ± 41.77 ^c^	1770.25 ± 79.99 ^a^	1570.34 ± 40.16 ^b^
42	(+)-Limonene-M	138-86-3	1200.9	483.337	2582.86 ± 58.57 ^c^	2704.58 ± 18.98 ^c^	1860.12 ± 30.30 ^d^	2085.54 ± 26.63 ^d^
43	(+)-Limonene-D	138-86-3	1199.1	481.176	3064.01 ± 28.51 ^c^	3464.96 ± 23.87 ^b^	2363.66 ± 21.29 ^d^	2321.69 ± 49.50 ^d^
45	α-Terpinene-M	99-86-5	1173.9	452.001	2145.84 ± 141.61 ^c^	2221.87 ± 45.83 ^c^	3290.74 ± 47.68 ^a^	2660.70 ± 59.05 ^b^
46	δ-3-Carene	13466-78-9	1157.8	433.631	700.21 ± 28.66 ^b^	1242.64 ± 21.70 ^a^	512.93 ± 14.59 ^c^	556.88 ± 19.49 ^c^
48	α-Terpinene-D	99-86-5	1173.9	452.001	156.54 ± 27.67 ^b^	159.30 ± 12.11 ^b^	727.29 ± 48.03 ^a^	314.56 ± 37.58 ^b^
55	β-Pinene-M	127-91-3	1119.3	392.707	1960.20 ± 43.48 ^b^	2182.71 ± 41.55 ^a^	2103.62 ± 16.54 ^a^	2040.80 ± 23.65 ^a^
56	β-Pinene-D	127-91-3	1118.8	392.196	403.06 ± 52.90 ^c^	1452.75 ± 39.33 ^a^	582.83 ± 30.12 ^c^	552.95 ± 38.60 ^c^
62	α-Pinene-M	80-56-8	1025.9	316.521	1473.00 ± 23.88 ^a^	1695.04 ± 31.93 ^a^	1022.68 ± 6.05 ^c^	1127.33 ± 7.81 ^b^
63	α-Pinene-D	80-56-8	1024.4	315.461	398.20 ± 43.90 ^c^	1112.18 ± 62.55 ^a^	279.95 ± 17.40 ^d^	297.18 ± 16.36 ^d^
69	α-Pinene-T	80-56-8	1023.6	314.931	173.33 ± 17.76 ^c^	495.59 ± 37.17 ^b^	131.39 ± 9.10 ^c^	117.43 ± 12.73 ^c^

Aldehydes								
12	(E)-2-Octenal-M	25488-70-4	1429.6	933.939	278.64 ± 10.99 ^b^	235.18 ± 13.46 ^b^	996.25 ± 75.45 ^a^	607.37 ± 39.38 ^a^
13	(E)-2-Octenal-D	25488-70-4	1429.1	932.57	56.41 ± 9.96 ^a^	64.21 ± 9.75 ^a^	121.87 ± 13.17 ^a^	65.32 ± 6.40 ^a^
15	2-Furaldehyde	98-01-1	1477.1	1085.821	851.78 ± 143.88 ^a^	433.91 ± 22.90 ^b^	209.06 ± 6.75 ^c^	199.34 ± 35.14 ^c^
17	1-Nonanal	124-19-6	1396.9	841.97	663.02 ± 37.30 ^a^	570.54 ± 10.54 ^a^	593.12 ± 27.68 ^a^	702.61 ± 22.48 ^a^
18	(E,E)-2,4-Hexadienal	142-83-6	1404.6	862.797	77.66 ± 8.11 ^a^	63.49 ± 8.03 ^a^	194.72 ± 13.72 ^a^	181.64 ± 3.89 ^a^
23	2,6-Dimethyl-5-heptenal	106-72-9	1354.1	735.231	769.65 ± 90.55 ^b^	879.74 ± 24.66 ^b^	718.38 ± 9.28 ^b^	1553.13 ± 37.84 ^a^
26	(E)-2-Heptenal-M	18829-55-5	1327.8	676.22	784.57 ± 13.50 ^a^	644.13 ± 8.54 ^b^	878.17 ± 18.82 ^a^	964.80 ± 16.54 ^a^
27	(E)-2-Heptenal-D	18829-55-5	1327.8	676.22	183.13 ± 11.47 ^a^	109.04 ± 12.66 ^b^	408.47 ± 36.11 ^a^	429.68 ± 1.43 ^a^
32	1-Octanal	124-13-0	1297.9	615.165	214.07 ± 29.37 ^a^	167.42 ± 4.64 ^a^	246.62 ± 19.24 ^a^	254.62 ± 7.73 ^a^
34	(Z)-4-Heptenal	67283-10-9	1255	551.952	666.94 ± 81.33 ^a^	616.97 ± 51.91 ^a^	796.31 ± 77.89 ^a^	830.03 ± 66.21 ^a^
40	(E)-2-Hexen-1-al-M	67282-63-0	1220.9	507.649	1146.95 ± 3.20 ^a^	760.50 ± 123.78 ^b^	1149.78 ± 20.88 ^a^	1240.05 ± 14.39 ^a^
41	(E)-2-Hexen-1-al-D	67282-63-0	1221.3	508.19	1255.33 ± 12.17 ^c^	454.13 ± 128.07 ^d^	6636.95 ± 49.46 ^a^	6358.02 ± 97.44 ^a^
53	(E)-2-Pentenal-M	1576-87-0	1134.4	408.238	666.48 ± 16.95 ^a^	701.82 ± 12.41 ^a^	597.12 ± 11.69 ^b^	615.79 ± 3.59 ^b^
54	(E)-2-Pentenal-D	1576-87-0	1132.8	406.617	273.05 ± 12.03 ^b^	166.77 ± 12.56 ^c^	1109.27 ± 60.66 ^a^	528.69 ± 5.66 ^b^
57	1-Hexanal-D	66-25-1	1088	362.302	2256.74 ± 64.33 ^b^	2032.99 ± 27.04 ^b^	2441.10 ± 39.98 ^a^	2744.00 ± 263.86 ^a^
65	n-Pentanal	110-62-3	991.5	293.727	1335.59 ± 29.25 ^b^	1585.65 ± 40.21 ^a^	1207.54 ± 44.22 ^b^	1522.96 ± 43.05 ^a^
70	1-Hexanal-M	66-25-1	1088.6	362.903	432.85 ± 13.26 ^a^	468.46 ± 23.99 ^a^	185.94 ± 18.59 ^b^	181.74 ± 9.79 ^b^
73	3-Methyl butanal	590-86-3	928.7	260.067	2660.89 ± 35.95 ^b^	3143.05 ± 38.02 ^a^	2495.02 ± 33.93 ^c^	2326.01 ± 25.99 ^c^
75	2-Methyl-2-propenal	78-85-3	898.1	245.224	2174.54 ± 11.87 ^b^	2219.42 ± 22.24 ^b^	1926.98 ± 9.47 ^c^	2016.97 ± 7.49 ^c^
77	Propanal	123-38-6	840.5	219.515	1252.15 ± 12.87 ^a^	1188.62 ± 9.92 ^a^	1456.27 ± 15.46 ^a^	1333.55 ± 10.71 ^a^
87	1,1-Diethoxy ethane	105-57-7	908.9	250.333	375.92 ± 58.68 ^b^	351.16 ± 28.38 ^b^	977.61 ± 31.03 ^a^	802.46 ± 9.21 ^a^

Esters								
2	Linalyl acetate	115-9-75	1573.3	1473.433	81,526.40 ± 2439.25 ^b^	31,358.02 ± 3520.22 ^a^	34,703.22 ± 4151.52 ^a^	32,920.76 ± 3492.80 ^a^
71	Ethyl 2-methy lpropionate	97-62-1	956.6	274.379	1678.92 ± 22.91 ^a^	1533.97 ± 11.10 ^a^	1733.23 ± 46.61 ^a^	1593.02 ± 68.25 ^a^
79	(Z)-3-Hexen-1-yl acetate	3681-71-8	1325.7	671.884	323.61 ± 15.07 ^a^	318.00 ± 13.66 ^a^	271.35 ± 8.72 ^a^	299.09 ± 11.67 ^a^
84	sec-Butyl acetate	105-46-4	1009.3	305.3	978.67 ± 65.20 ^b^	1997.98 ± 99.40 ^a^	700.84 ± 23.05 ^b^	542.23 ± 30.01 ^b^
85	Acetic acid ethyl ester	141-78-6	879	236.356	876.40 ± 25.47 ^a^	738.36 ± 7.46 ^b^	962.23 ± 8.71 ^a^	832.15 ± 4.86 ^a^

Ketones								
3	d-Camphor-M	464-49-3	1551.2	1373.476	3018.09 ± 319.97 ^b^	2395.63 ± 116.13 ^c^	4384.05 ± 35.82 ^a^	3792.20 ± 172.02 ^a^
4	d-Camphor-D	464-49-3	1550.6	1370.738	954.97 ± 126.92 ^b^	792.78 ± 44.72 ^b^	2366.20 ± 164.83 ^a^	2008.48 ± 86.16 ^a^
6	Isomenthone-M	491-07-6	1493.8	1144.807	6385.67 ± 716.42 ^b^	5591.47 ± 70.39 ^c^	5776.16 ± 130.53 ^c^	5675.39 ± 265.26 ^c^
7	Isomenthone-D	491-07-6	1493.8	1144.807	19,881.10 ± 1159.39 ^a^	18,638.00 ± 260.02 ^a^	20,862.75 ± 1014.41 ^a^	15,798.70 ± 823.20 ^b^
8	2-Decanone	693-54-9	1494.9	1148.915	7723.96 ± 5620.15 ^b^	15,970.24 ± 547.25 ^a^	16,068.01 ± 1171.88 ^a^	15,458.15 ± 2323.41 ^a^
24	2-Methyl-2-hepten-6-one	110-93-0	1343.9	711.8	2180.49 ± 11.44 ^d^	2708.08 ± 71.21 ^c^	3425.77 ± 108.72 ^a^	3473.29 ± 43.05 ^a^
60	2,3-Pentadione	60-01-4	1065.1	344.615	4026.26 ± 212.31 ^b^	6065.31 ± 228.47 ^a^	4112.60 ± 258.64 ^b^	3368.27 ± 379.09 ^b^
61	1-Penten-3-one	1629-58-9	1030.1	319.436	529.63 ± 22.43 ^b^	242.15 ± 2.74 ^c^	2387.71 ± 71.02 ^a^	1356.15 ± 59.28 ^b^
64	4-Methyl-2-pentanone	108-10-1	1013.4	308.039	357.58 ± 7.38 ^a^	303.60 ± 8.84 ^b^	311.46 ± 3.16 ^b^	385.05 ± 3.57 ^a^
66	2-Pentanone	107-87-9	987.4	291.077	426.03 ± 4.02 ^b^	358.36 ± 17.43 ^c^	1055.31 ± 86.84 ^a^	570.77 ± 49.59 ^b^
67	2,3-Butanedione	431-03-8	975.4	284.451	424.27 ± 12.50 ^b^	546.56 ± 39.69 ^a^	536.85 ± 9.14 ^a^	557.22 ± 14.26 ^a^
72	2-Pentanone	107-87-9	941.3	266.428	1174.99 ± 3.77 ^b^	1123.70 ± 38.79 ^b^	1426.76 ± 46.14 ^a^	1357.74 ± 34.01 ^a^
74	2-Butanone	78-93-3	914.8	253.176	1649.90 ± 27.08 ^a^	1820.74 ± 44.70 ^a^	1641.73 ± 59.49 ^a^	1669.97 ± 64.99 ^a^
76	2-Propanone	67-64-1	857.8	226.937	4420.75 ± 26.79 ^b^	4199.87 ± 81.07 ^b^	4387.82 ± 68.68 ^b^	4867.40 ± 36.29 ^a^
80	1-Octen-3-one	4312-99-6	1313.3	645.845	308.16 ± 19.34 ^c^	941.78 ± 37.96 ^a^	461.51 ± 27.33 ^b^	269.78 ± 25.17 ^c^
86	Acetone	67-64-1	823.2	212.384	1049.59 ± 10.23 ^c^	1266.75 ± 21.57 ^b^	1250.75 ± 12.72 ^b^	1410.16 ± 15.10 ^a^

Alkanes								
82	Undecane-M	1120-21-4	1102.4	376.008	414.07 ± 8.14 ^a^	312.41 ± 9.07 ^b^	302.86 ± 4.06 ^b^	267.90 ± 5.88 ^c^
83	Undecane-D	1120-21-4	1101.7	375.331	624.68 ± 15.32 ^a^	479.88 ± 42.02 ^b^	859.82 ± 99.43 ^a^	681.07 ± 68.95 ^a^

Aromatic								
5	Benzaldehyde	100-52-7	1522.9	1255.719	909.35 ± 88.60 ^b^	627.92 ± 32.91 ^c^	2603.22 ± 199.41 ^a^	1114.61 ± 58.05 ^b^
9	2,6-Dimethyl-3-ethylpyrazine	13925-07-0	1474.7	1077.713	739.53 ± 135.36 ^c^	1139.45 ± 57.26 ^a^	1111.61 ± 114.10 ^a^	991.50 ± 78.84 ^b^
44	1,2-Dimethylbenzene	95-47-6	1184.2	463.887	2102.88 ± 18.99 ^a^	2078.22 ± 12.20 ^a^	1852.79 ± 39.52 ^b^	2072.63 ± 36.98 ^a^
50	1-(1-methylethyl)-4-Methylbenzene	99-87-6	1284.9	594.094	154.49 ± 11.81 ^b^	154.40 ± 4.49 ^b^	516.10 ± 44.06 ^a^	235.86 ± 22.82 ^b^

Others								
14	Acetic acid	64-19-7	1479.3	1093.632	612.53 ± 163.08 ^a^	429.29 ± 33.86 ^a^	477.42 ± 90.15 ^a^	524.31 ± 53.09 ^a^
16	1,8-Cineole	470-82-6	1420.1	906.187	233.61 ± 11.25 ^b^	273.85 ± 38.83 ^b^	905.92 ± 89.00 ^a^	742.33 ± 186.60 ^a^
28	3-Mercapto-2-butanone-M	40789-98-8	1321.6	663.203	1008.14 ± 25.88 ^a^	644.30 ± 22.04 ^b^	1176.89 ± 7.43 ^a^	1117.24 ± 26.62 ^a^
29	3-Mercapto-2-butanone-D	40789-98-8	1320.8	661.468	283.61 ± 16.17 ^b^	125.11 ± 2.15 ^c^	587.27 ± 26.36 ^a^	445.91 ± 8.54 ^a^
37	Thiazole	288-47-1	1251.8	547.63	432.13 ± 14.05 ^a^	371.84 ± 5.39 ^b^	282.63 ± 7.33 ^c^	408.04 ± 6.57 ^a^
78	4,5-Dimethylthiazole	35819-17-1	1376.6	789.563	406.77 ± 9.24 ^a^	234.04 ± 7.74 ^c^	316.59 ± 25.95 ^b^	275.05 ± 17.77 ^bc^

Values represent the peak-volume responses obtained from GC–IMS and are expressed as arbitrary units (a.u.). Different lowercase letters within the same row indicate significant differences among drying treatments according to one-way ANOVA followed by Duncan's multiple range test (*P* < 0.05).

The detailed sensitivity characteristics of the PEN3 electronic nose sensors are summarized in Table S2. The response profiles of the ten sensors are presented in Fig. 1C. W2W showed the highest response intensity across the four drying treatments, indicating that compounds associated with aromatics, sulfides, and related volatile components may contribute substantially to the overall aroma profiles. W2S also exhibited relatively strong responses, suggesting contributions from alcohols, aldehydes, and ketones. In addition, differences in the response intensities of W1C, W5S, W6S, and W5C were observed among the drying treatments, indicating that drying conditions altered the overall sensor response patterns rather than affecting only a single class of volatile compounds.

PCA based on *E*-nose responses showed distinct distribution patterns among the four drying treatments (Fig. 1D). PC1 and PC2 explained 56.6% and 19.7% of the total variance, respectively, accounting for 76.3% cumulatively. The three biological replicates within each treatment showed relatively consistent clustering, while the four drying treatments were distributed in different regions of the PCA score plot. HAD samples were located in the negative region of PC1, whereas MD and ND samples were distributed mainly in the positive region of PC1. VFD samples were positioned separately from the other treatments, mainly in the negative PC1 and PC2 regions. These results indicate that the four drying treatments produced distinguishable overall E-nose response patterns.

Overall, the E-nose analysis revealed treatment-associated differences in the sensor response profiles of dried Chuju, with relatively consistent responses among biological replicates within each treatment. The distinct E-nose profiles provide additional evidence for differences in the overall aroma characteristics induced by the four drying methods and support the subsequent characterization of volatile compounds by GC–IMS.

### 3.2 Analysis of volatile compounds in dried Chuju using different methods

Volatile profiles of dried Chuju samples were characterized using GC–IMS. A total of 90 volatile features were tentatively identified based on retention indices, drift times, and database matching, including alcohols, terpenes, aldehydes, esters, ketones, alkanes, aromatic compounds, and other volatile compounds (Table 1). Based on relative peak volume differences, tentative annotation results, and consistency with sensory evaluation, representative volatile features were selected for comparative interpretation. Linalyl acetate, isomenthone, 2-decanone, 2-propanone, 2,3-pentanedione, *γ*-terpinene, (E)-2-hexenal, and d-camphor showed relatively high peak volumes, and their relative abundances differed significantly among drying treatments (*P* < 0.05, Table 1).

The two-dimensional GC–IMS topographic plots showed distinct differences among drying treatments (Fig. 2A). Most volatile signals were distributed within retention times of 200–800 s and drift times of 1.0–1.8 ms. HAD exhibited relatively stronger overall signal intensity compared with other treatments, suggesting that thermal drying substantially modified volatile profiles. Similar increases in aldehydes and ketones after hot-air drying have been reported in Gongju and other chrysanthemum products, which were mainly attributed to thermal transformation of endogenous precursors (Sun et al., 2026; Wang, Li, et al., 2025).

**Fig. 2:**
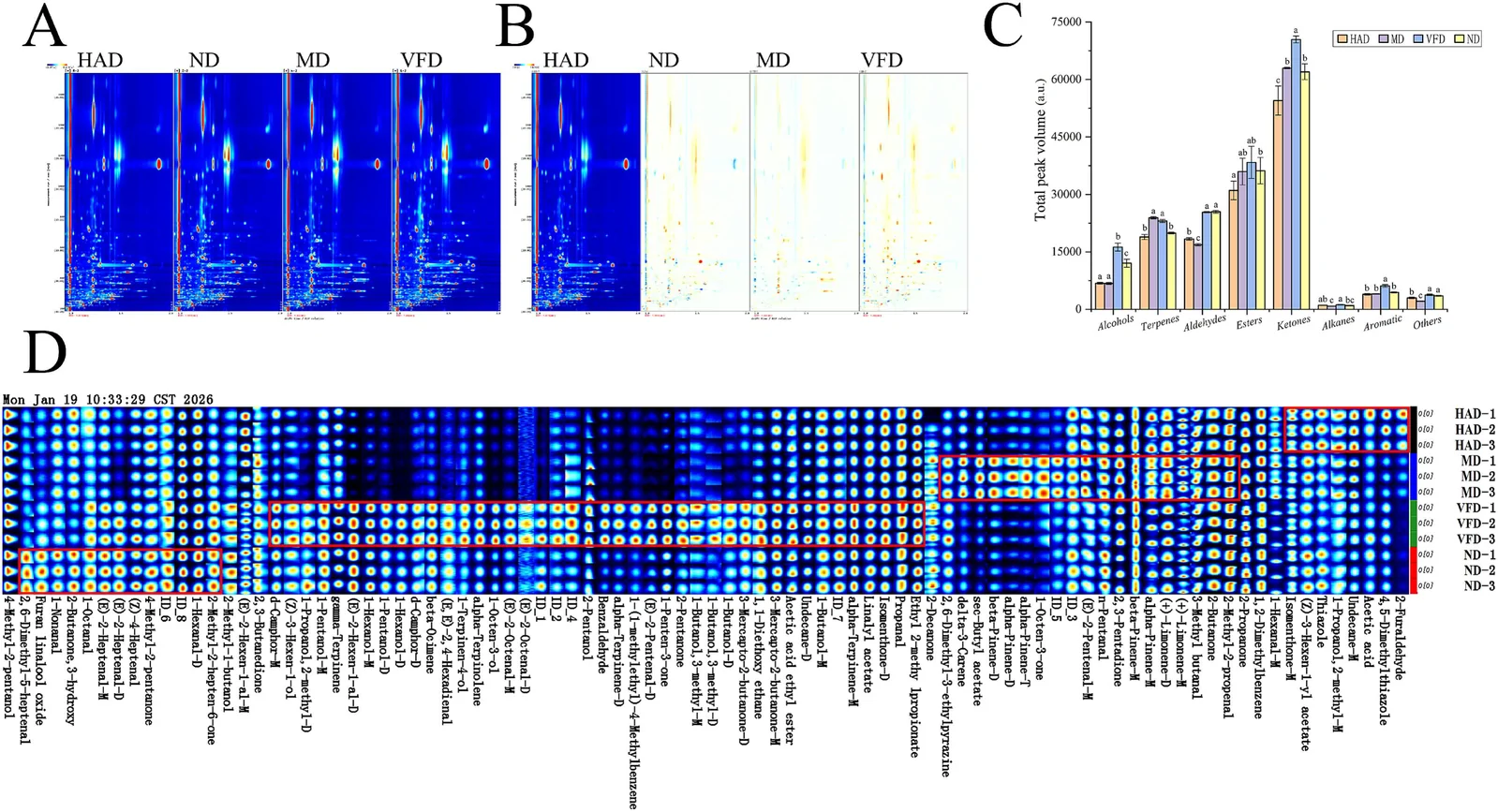
Effects of different drying methods on volatile organic compounds (VOCs) profiles of Chuju analyzed by gas chromatography-ion mobility spectrometry (GC-IMS). (A) Two-dimensional topographic plots of VOCs in Chuju samples.(B) Differential comparison spectra of VOCs with HAD as the reference group. (C) Total peak volumes of major VOC categories. (D) GC-IMS fingerprint patterns of volatile components in different drying groups. Data are presented as mean ± SD (*n* = 3). a.u., arbitrary units of GC–IMS instrumental response. Different lowercase letters above the bars within the same VOCs category indicate significant differences among drying methods (*P* < 0.05, one-way ANOVA followed by Duncan's multiple range test).

Differential comparison spectra using HAD as the reference further revealed treatment-specific volatile variations (Fig. 2B). Compared with HAD, VFD showed increased signals for several volatile regions, particularly those associated with oxygen-containing compounds. These differences suggest that low-temperature drying may favor the retention of certain volatile precursors compared with thermal treatments.

Relative quantification based on GC–IMS peak volumes revealed differences in volatile categories among drying treatments (Fig. 2C). Aldehydes and ketones represented major volatile classes after drying, indicating that dehydration processes altered precursor conversion pathways. Similar changes from terpene/alcohol-dominated profiles in fresh chrysanthemum flowers toward aldehyde- and ketone-enriched profiles after drying have been reported in Hangbaiju and Gongju (Shi et al., 2017; Zhang et al., 2025). These changes may result from combined effects of lipid oxidation, cell disruption, and thermal transformation during dehydration.

VFD samples showed relatively higher levels of several aroma-associated volatile compounds, including (*E*)-2-hexenal, terpinen-4-ol, and 1-octen-3-ol (Table 1). These compounds have previously been associated with green and floral aroma characteristics (Fan and Liu, 2025; Wang et al., 2019). Their higher relative abundance in VFD may partly explain the stronger floral and green-grass sensory attributes observed in Fig. 1B. HAD samples showed higher signal intensities of compounds such as 2,3-pentanedione and 3-methylbutanal. These compounds are commonly associated with sweet and roasted aroma notes and may originate from amino acid and carbohydrate-related transformations during heating. Similar accumulation patterns have been observed during hot-air drying of chrysanthemum varieties (Sun et al., 2026). MD samples exhibited relatively higher levels of (+)-limonene-D and 2-methyl-2-propenal, which may contribute to their intermediate aroma characteristics. ND samples showed higher relative abundance of some aldehydes, including n-pentanal and propanal, which may be related to prolonged exposure to oxygen during drying.

Compared with previous studies reporting 65 VOCs in fresh Chuju flowers (Zhang et al., 2025), the present study identified 90 VOCs in dried Chuju, indicating that drying substantially modified the volatile composition. Previous studies suggested that alcohols and terpenes were dominant volatile classes in fresh Chuju (Gao et al., 2025; Zhang et al., 2025), whereas ketones and aldehydes showed higher relative contributions after drying. This difference may be attributed to the combined effects of thermal exposure, oxygen availability, and drying duration on volatile precursor transformation (Chen et al., 2025; Shi et al., 2026; Zeng et al., 2026). However, the specific metabolic mechanisms underlying these VOC alterations were further investigated through metabolomic analysis.

Overall, GC–IMS analysis demonstrated that drying treatments significantly modified the volatile fingerprints of Chuju. VFD mainly favored the retention of floral-associated volatile compounds, whereas HAD was associated with higher levels of thermal-associated volatile compounds. MD and ND showed intermediate volatile profiles, reflecting differences in heat transfer and drying environment.

### 3.3 Metabolite analysis and metabolic differences between groups

Metabolomic profiling of dried Chuju samples was conducted using both positive and negative electrospray ionization modes. Representative total ion current chromatograms are shown in Fig. S4A and B. After peak identification, retention time alignment, and data filtering, a total of 61,330 valid characteristic features were extracted. Based on matching with the updated local database using retention time (RT), precursor ion mass (MS1), and MS/MS fragmentation information (MS2), 2605 metabolites were annotated. According to the MSI-referenced identification criteria used by the metabolomics platform, the annotated metabolites were classified into different confidence levels. Specifically, 987 metabolites were assigned as Level 1, based on matching with authentic standards in MS1, MS2, and RT; 1169 metabolites were assigned as Level 2, based on MS1 and MS2 matching with public databases; and 449 metabolites were assigned as Level 3, based on matching of MS1, MS2, and RT information without authentic standard confirmation. The remaining 58,725 unknown features were classified as Level 4. According to the HMDB superclass classification (Fig. S4C and D), the identified metabolites were assigned to 10 major superclasses and 55 subclasses. Phenylpropanoids represented the largest superclass (29.93%), followed by lipids (20.62%), terpenoids (17.89%), and alkaloids (10.27%). The high proportion of phenylpropanoid-, lipid-, and terpenoid-related metabolites indicates that these metabolic categories represent important components of the Chuju metabolome. Phenylpropanoid derivatives, including flavonoids and phenolic compounds, are characteristic secondary metabolites in chrysanthemum species and contribute to antioxidant activity and quality attributes. Terpenoid-related metabolites are closely associated with floral and herbal aroma characteristics, whereas lipid-derived metabolites can serve as precursors for volatile formation during dehydration. Therefore, changes in these metabolite classes may contribute to aroma and quality differences among drying treatments.

Similar metabolite categories have also been reported in other chrysanthemum cultivars, including Gongju, Hangbaiju, and Boju, where phenylpropanoids, flavonoids, and terpenoids represent important metabolic components associated with functional properties and aroma characteristics (Shi et al., 2017; Wang et al., 2019). However, the relative composition and response to drying conditions may vary among cultivars due to differences in genotype and initial chemical composition. Considering the characteristic aroma profile of Chuju, the changes in terpenoid- and volatile precursor-related metabolites may have important implications for aroma differentiation during drying.

PCA was first performed to visualize the overall distribution patterns of the metabolomic profiles among the four drying treatments (Fig. S4E). PC1 and PC2 explained 32.1% and 22.1% of the total variance, respectively, accounting for 54.2% cumulatively. Because the first two principal components did not capture the complete metabolic variation, a three-dimensional PCA was additionally performed. PC3 explained a further 12.6% of the total variance, and the first three principal components collectively accounted for 66.8% of the total variance (Fig. S4F). Samples from the same drying treatment showed relatively tight clustering, whereas the four drying treatments exhibited distinct distribution patterns, indicating treatment-associated differences in the overall metabolic profiles. VFD and HAD samples were located at opposite positions along PC1, while ND samples showed a distinct distribution from the other drying treatments. These PCA results provide an overview of the major distribution patterns of the metabolomic profiles rather than a complete representation of all metabolic variation.

The stability and reproducibility of the metabolomic measurements were further assessed using the PCA-based quality-control analysis. The one-dimensional distributions of PC1 and PC2 showed that the QC and experimental samples were within the ±2 SD ranges (Fig. S4G and H), and the QC samples were closely clustered in the PCA score plot (Fig. S4I), indicating good analytical stability and reproducibility. Taken together, the PCA and QC analyses supported the consistency of the metabolomic dataset and provided an overview of the metabolic differences associated with the four drying treatments. These differences were subsequently characterized in greater detail by differential metabolite analysis and pathway enrichment analysis.

The potential overfitting of the supervised discrimination models was further evaluated using OPLS-DA permutation tests (Fig. S5). The negative Q^2^ intercepts obtained from the permutation tests indicated that the original models performed better than the corresponding permuted models, suggesting no evident overfitting. These results provided additional support for the robustness of the supervised discrimination analysis among the drying treatments.

### 3.4 Screening of differential metabolites

To characterize metabolic response patterns induced by different drying methods, K-means clustering analysis was performed based on differential metabolite abundance profiles. The optimal cluster number was determined using both the Elbow Method and Silhouette Coefficient (Fig. S3). Although the elbow curve indicated a possible inflection at k = 4, the highest silhouette coefficient and better biological interpretability were obtained at k = 3. Therefore, three clusters were selected for subsequent analysis. As shown in Fig. S6, differential metabolites exhibited distinct accumulation patterns among HAD, ND, VFD, and MD treatments.

Cluster 1 contained 618 metabolites with relatively higher abundance in ND samples. These metabolites included several oxidation-related and stress-responsive compounds (Fig. 3E and F). The enrichment of these metabolites under ND conditions may reflect metabolic responses associated with prolonged drying duration and environmental exposure. Compared with controlled drying methods, natural drying provides a longer period for oxygen exposure and may increase the occurrence of oxidation-related metabolic changes. Similar observations have been reported in naturally dried chrysanthemum products, where extended drying duration influenced phenolic metabolism and volatile composition (Fan et al., 2025; Wang, Li, et al., 2025).

**Fig. 3:**
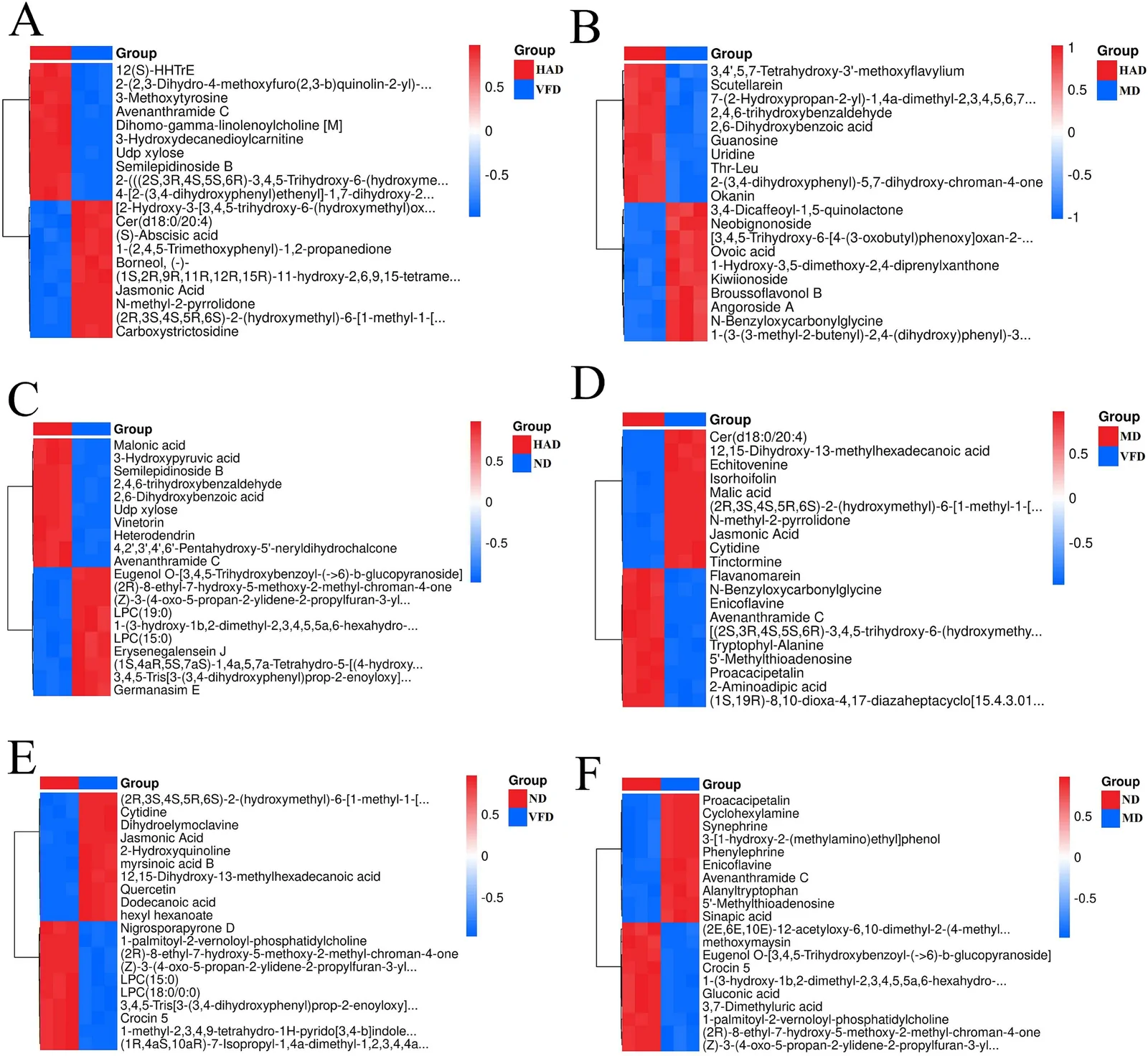
Hierarchical clustering heatmap of top differential metabolites in each pairwise comparison (*P* < 0.05). (A) HAD vs. VFD, (B) HAD vs. MD, (C) HAD vs. ND, (D) MD vs. VFD, (E) ND vs. VFD, (F) ND vs. MD. Red = high relative abundance; blue = low relative abundance. (For interpretation of the references to color in this figure legend, the reader is referred to the web version of this article.)

Cluster 2 contained 863 metabolites and showed higher abundance in HAD samples, followed by MD. These metabolites were mainly associated with amino acid derivatives, phenolic-related compounds, and thermal-responsive metabolic pathways (Fig. 3A–C). The accumulation pattern in HAD samples was consistent with the higher abundance of aldehydes and ketones observed in GC–IMS analysis. During hot-air drying, elevated temperature may be associated with enhanced transformation of amino acid- and carbohydrate-derived precursors, which may contribute to differences in thermally associated aroma compounds. Similar thermal-induced metabolic changes have been reported in Gongju and Jinsihuangju during drying processes (Qian et al., 2025; Shi et al., 2017; Sun et al., 2026).

Cluster 3 contained 691 metabolites and exhibited relatively higher abundance in VFD samples. Several representative metabolites, including flavonoid-related compounds, caffeoylquinic acid derivatives, and lysophosphatidylcholine (LPC)-related metabolites, showed higher retention under VFD conditions (Fig. 3A, D, and E). These results suggest that low-temperature drying may favor the preservation of some thermolabile metabolites compared with thermal drying. Similar retention effects have been reported in chrysanthemum and other plant materials subjected to freeze-drying (Han et al., 2024).

Differential metabolites were further screened using VIP > 1, *P* < 0.05, and FC ≥ 2 or FC ≤ 0.5 as criteria. Volcano plots of six pairwise comparisons are shown in Fig. S7A–F. The largest number of differential metabolites was observed between HAD and VFD, with 1476 metabolites identified (696 upregulated and 780 downregulated), indicating the greatest metabolic divergence between thermal drying and vacuum freeze-drying. The remaining comparisons included 1129 metabolites for HAD vs. MD, 1352 for HAD vs. ND, 1212 for ND vs. MD, 1288 for ND vs. VFD, and 1428 for MD vs. VFD (Table 2).

**Table 2:** Numbers and regulation patterns of differential metabolites identified in pairwise comparisons among Chuju samples subjected to different drying methods.

Comparison	Total	Up-regulated	Down-regulated
HAD vs VFD	1476	696	780
HAD vs MD	1129	370	759
HAD vs ND	1352	526	826
ND vs MD	1212	553	659
ND vs VFD	1288	706	582
MD vs VFD	1428	806	622

Differential metabolites were screened using VIP > 1, *P* < 0.05, and FC ≥ 2 or FC ≤ 0.5. Up- and down-regulated metabolites were determined according to the fold-change direction in each pairwise compariso.

The representative differential metabolites identified in the pairwise comparisons belonged to multiple metabolic categories, including phenylpropanoids, lipids, terpenoids, amino acids and their derivatives, carbohydrates and nucleosides, organic acids, alkaloids, and polyketides (Table S3). Notably, the HAD vs. VFD comparison showed extensive changes in lipid- and terpenoid-related metabolites, with jasmonic acid, ceramide [Cer(d18:0/20:4)], borneol, and several monoterpenoid and sesquiterpenoid compounds among the representative differential metabolites. In contrast, several phenolic acids and amino acid derivatives, such as 3-methoxytyrosine and 2,6-dihydroxybenzoic acid, were decreased in this comparison. The HAD vs. MD comparison was characterized by pronounced changes in nucleosides and phenylpropanoid-related metabolites, including decreased uridine, guanosine, scutellarein, and 2,6-dihydroxybenzoic acid, together with increased levels of several phenylpropanoid and flavonoid-related metabolites. The HAD vs. ND comparison similarly involved substantial changes in lipids, phenylpropanoids, organic acids, and terpenoids, with jasmonic acid and several lysophosphatidylcholine species showing increased levels, whereas malonic acid and several phenolic compounds were decreased. VFD vs. MD and ND vs. VFD comparisons also revealed extensive alterations in flavonoids, lipids, amino acid derivatives, nucleosides, and terpenoids. For example, quercetin was increased in ND vs. VFD, while jasmonic acid and several lipid-related metabolites were differentially regulated across comparisons. Collectively, these results indicate that drying treatments induced broad changes in both primary and secondary metabolism, with phenylpropanoid/flavonoid, lipid, and terpenoid metabolism representing prominent metabolic features distinguishing the drying treatments.

Overall, differential metabolite analysis demonstrated that different drying methods were associated with distinct metabolic response patterns. HAD was mainly characterized by thermal-associated metabolic changes, including increased accumulation of amino acid- and carbohydrate-related metabolites potentially involved in aroma precursor transformation. VFD showed greater retention of several secondary metabolites, including flavonoid-related compounds, caffeoylquinic acid derivatives, and lysophosphatidylcholine-related metabolites. ND exhibited oxidation-associated metabolic responses, whereas MD displayed intermediate characteristics between thermal transformation and metabolite retention. These metabolic differences provide a basis for further pathway enrichment and metabolite–volatile correlation analyses.

### 3.5 KEGG pathway enrichment and metabolic mechanism analysis

To further explore the metabolic basis underlying aroma differences among drying treatments, KEGG enrichment and pathway topology analyses were performed based on differential metabolites identified from pairwise comparisons (Fig. 4, Fig. 5). Across different comparisons, differential metabolites were mainly associated with amino acid metabolism, central carbon metabolism, lipid-related metabolism, and secondary metabolite biosynthesis. These pathways represent important metabolic networks involved in precursor transformation and secondary metabolite variation during dehydration.

**Fig. 4:**
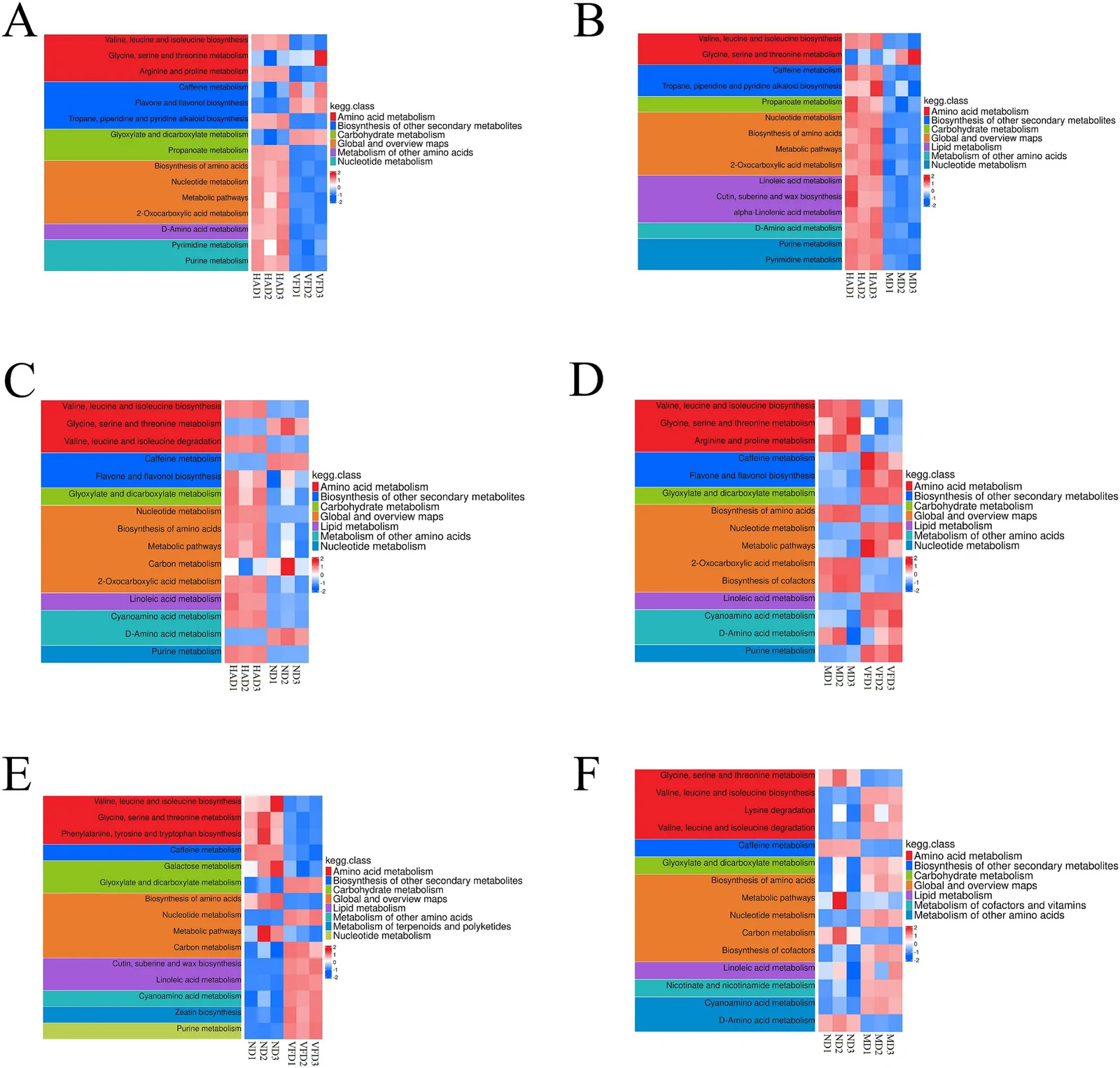
KEGG pathway enrichment heatmap of differentially accumulated metabolites between pairwise drying groups (*P* < 0.05). (A) HAD vs. VFD, (B) HAD vs. MD, (C) HAD vs. ND, (D) MD vs. VFD, (E) ND vs. VFD, (F) ND vs. MD. The x-axis shows samples; the y-axis shows enriched KEGG pathways. Color intensity represents the relative abundance of differential metabolites in each pathway (red = high, blue = low). (For interpretation of the references to color in this figure legend, the reader is referred to the web version of this article.)

**Fig. 5:**
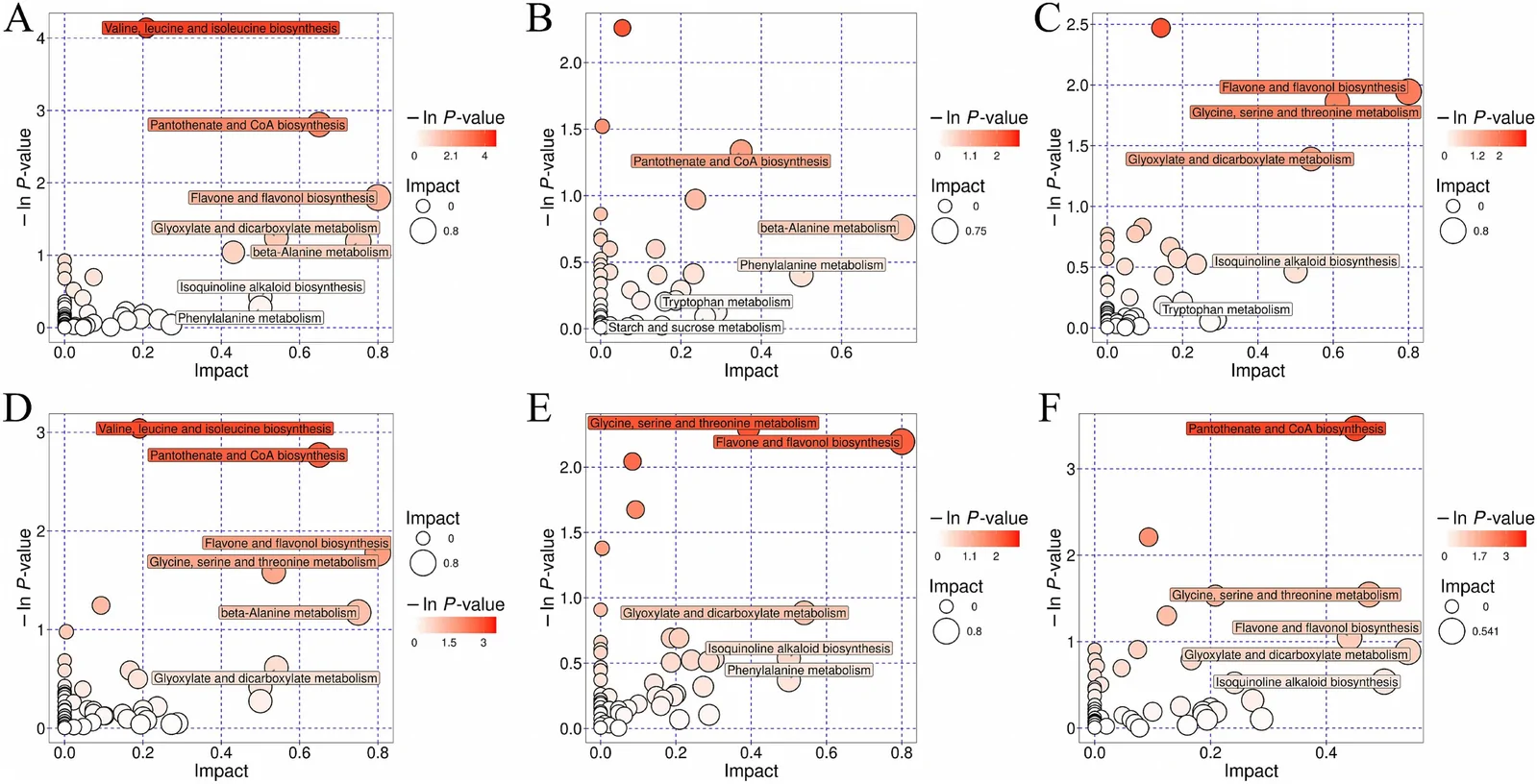
KEGG pathway topological analysis of differential metabolites in pairwise drying comparisons (*P* < 0.05). (A) HAD vs. VFD, (B) HAD vs. MD, (C) HAD vs. ND, (D) MD vs. VFD, (E) ND vs. VFD, (F) ND vs. MD. Bubble size indicates pathway impact factor; color depth represents −ln(P) (darker = more significant enrichment).

Among these pathways, amino acid metabolism showed frequent enrichment across comparisons, suggesting that drying-induced aroma differences may be related to changes in amino acid-derived precursor pools. In addition, repeated enrichment of lipid-related metabolism and phenylpropanoid/flavonoid biosynthesis indicated that membrane-associated metabolites and secondary metabolites were sensitive to different drying environments. These metabolic changes may contribute to the formation or retention of volatile compounds and functional metabolites during drying.

The HAD vs. VFD comparison exhibited the greatest metabolic divergence, which was consistent with their distinct aroma profiles observed by sensory evaluation and GC–IMS analysis. KEGG analysis indicated significant differences in pathways including 2-oxocarboxylic acid metabolism, amino acid biosynthesis, glycine, serine and threonine metabolism, and nucleotide metabolism (Figs. 4A and 5A). These pathways are closely associated with amino acid turnover and carbon skeleton metabolism. Because amino acids and reducing sugars are important precursors involved in thermal aroma formation (Yu et al., 2026), the altered abundance of amino acid-related metabolites under HAD conditions may be associated with the higher relative abundance of thermal-associated volatile compounds, including 2,3-pentanedione and 3-methylbutanal detected by GC–IMS. Similar associations between hot-air drying and enhanced amino acid-derived aroma formation have been reported in Gongju and Jinsihuangju (Shi et al., 2017; Sun et al., 2026). In contrast, VFD samples showed relatively higher abundance of several lipid-associated and secondary metabolite-related compounds. These metabolites may serve as precursors or intermediates associated with volatile formation and functional compound retention. The higher abundance of green and floral aroma-related volatiles, including (E)-2-hexenal, terpinen-4-ol, and 1-octen-3-ol, may therefore be related to improved preservation of aroma precursor metabolites under low-temperature drying conditions.

Although both HAD and MD involve thermal processing, their metabolic profiles differed substantially. The HAD vs. MD comparison showed enrichment of purine metabolism, nucleotide metabolism, lipid metabolism, linoleic acid metabolism, and cutin/suberin/wax biosynthesis pathways (Figs. 4B and 5B). These differences suggest that heating mode and dehydration rate may influence metabolic responses differently. Compared with conventional hot-air drying, microwave drying provides rapid internal heating, which may reduce prolonged thermal exposure. Similar advantages of microwave-assisted drying have been reported in Gongju, where shorter processing time contributed to improved retention of quality-related compounds (Wang et al., 2018).

For comparisons involving ND treatment, enriched pathways mainly included carbon metabolism, purine metabolism, glyoxylate and dicarboxylate metabolism, and amino acid metabolism (Figs. 4E, F, 5E, and F). These changes may reflect the influence of prolonged environmental exposure during natural drying. Compared with controlled drying processes, ND involves longer drying periods under variable temperature, humidity, and oxygen conditions, which may contribute to gradual metabolic rearrangement.

By integrating pathway enrichment, differential metabolites, and volatile profiles, three major metabolic processes potentially associated with aroma differentiation were identified. First, amino acid-related metabolism was closely associated with thermal aroma formation, particularly under HAD conditions, where differences in precursor-related metabolites were associated with higher abundance of thermal-associated volatiles. Second, lipid-related metabolism was associated with differences in green and floral aroma characteristics, as VFD samples showed relatively higher abundance of lipid-associated metabolites and related volatile compounds. Third, phenylpropanoid/flavonoid-related metabolism represented an important component of Chuju secondary metabolite variation during drying, particularly under low-temperature conditions.

Overall, these results suggest that different drying strategies influenced Chuju aroma through distinct metabolic responses rather than a single regulatory pathway. The integration of metabolomics and volatile profiling provides potential mechanistic explanations for aroma differentiation; however, further enzyme activity and targeted metabolite analyses are required to confirm the specific biochemical mechanisms involved.

### 3.6 Correlation analysis of differential metabolites and volatile compounds

To further explore the potential relationships between metabolic changes and volatile differences among drying treatments, Pearson correlation analysis was conducted between representative differential metabolites and GC–IMS-derived volatile compounds (Fig. S8). Differential metabolites were selected according to VIP > 1, *P* < 0.05, and FC ≥ 2 or ≤ 0.5, followed by KEGG annotation (Table S4). Metabolites related to aroma-associated pathways, including amino acid metabolism, phenylpropanoid biosynthesis, flavonoid biosynthesis, lipid metabolism, and carbohydrate metabolism, were selected for correlation analysis.

The HAD and VFD groups showed the most distinct metabolite–volatile correlation patterns (Fig. 6A). Several differential metabolites involved in amino acid metabolism showed strong correlations with floral and green aroma-related volatile compounds, including linalool, 1-terpinen-4-ol, and 1-octen-3-ol. Consistently, metabolic network analysis identified valine, leucine, and isoleucine biosynthesis (ath00290) as one of the pathways associated with metabolite differences between these two treatments (Fig. S8A). Considering that branched-chain amino acid metabolism provides potential precursors for volatile compounds, the distinct thermal environments of HAD and VFD may be associated with different metabolic profiles, with HAD exhibiting greater metabolic transformation whereas VFD showed enhanced retention of endogenous metabolites.

**Fig. 6:**
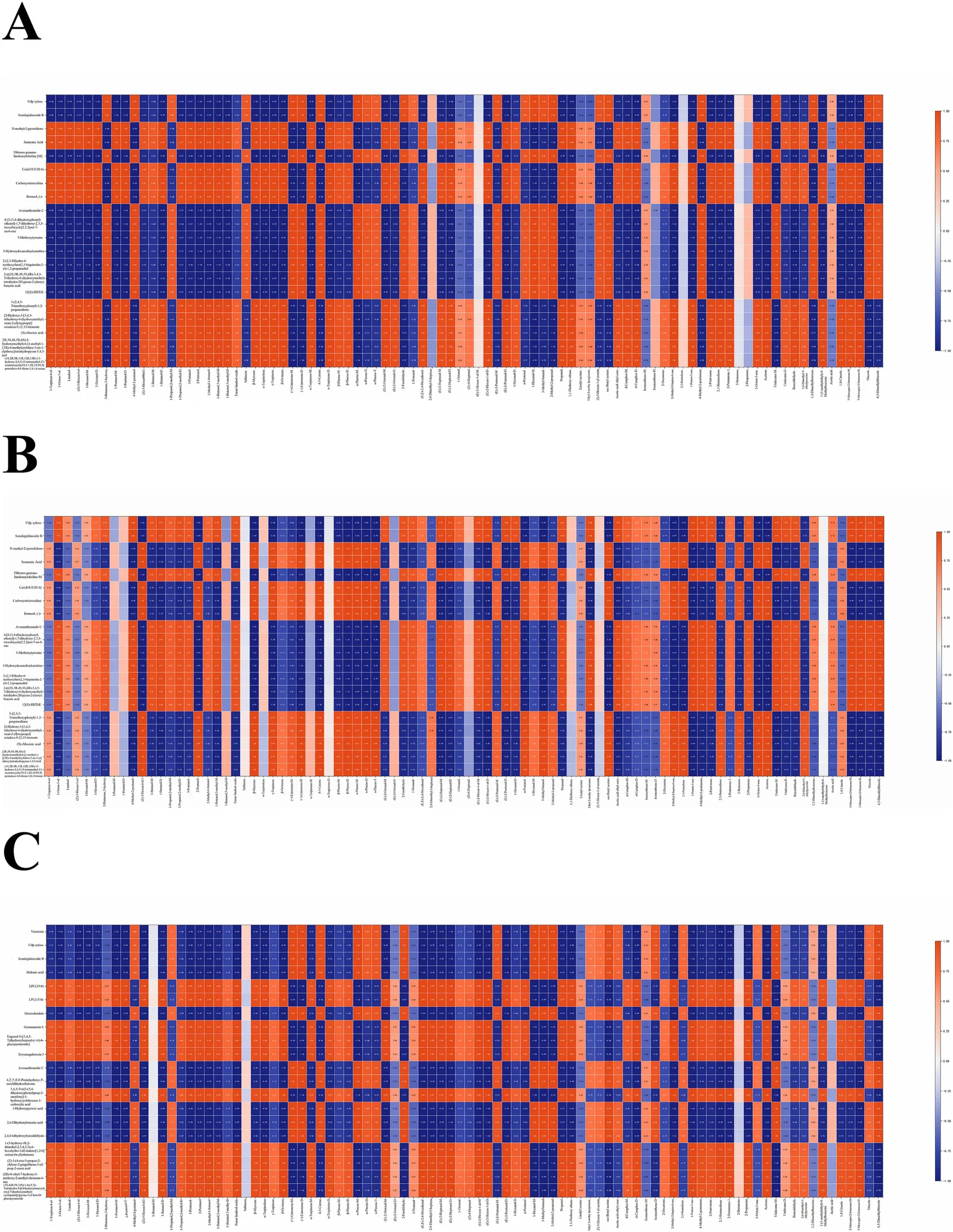

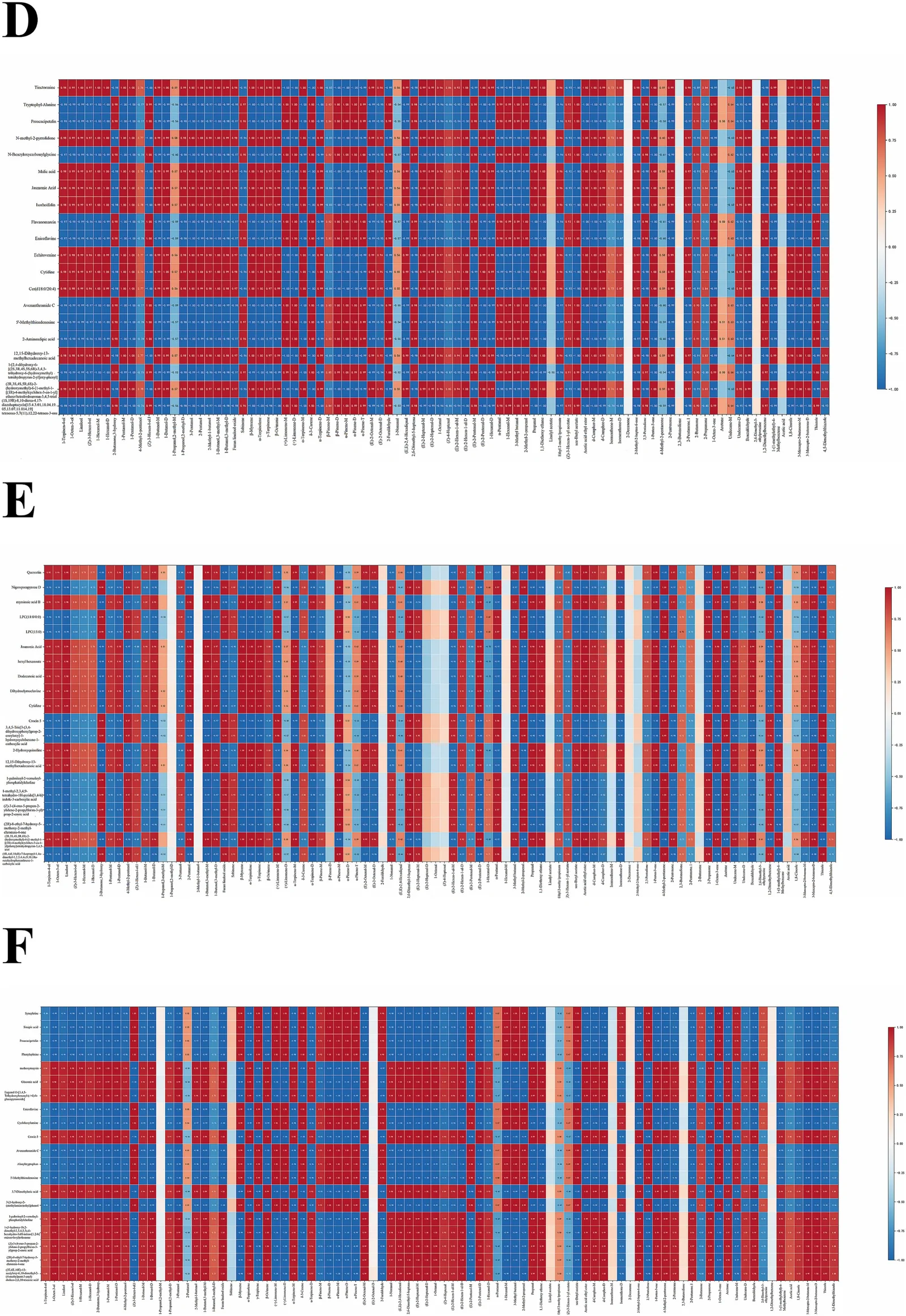
Pearson correlation analysis between representative differential metabolites and volatile compounds to explore potential aroma-associated metabolic changes. (A) HAD vs. VFD, (B) HAD vs. MD, (C) HAD vs. ND, (D) MD vs. VFD, (E) ND vs. VFD, (F) ND vs. MD. Red: positive correlation; blue: negative correlation; color intensity reflects correlation strength. (For interpretation of the references to color in this figure legend, the reader is referred to the web version of this article.)

The HAD and MD groups displayed distinguishable but relatively less divergent metabolite–volatile correlation patterns (Fig. 6B). Unlike the HAD–VFD comparison, the metabolic network did not reveal a single dominant pathway (Fig. S8B). Instead, multiple pathways associated with amino acid metabolism, lipid metabolism, and secondary metabolism collectively contributed to the observed differences. Differential metabolites involved in these pathways showed correlations with volatile compounds related to sweet and floral aroma characteristics. These results indicate that although both treatments involve thermal processing, differences in heat transfer mechanisms may induce distinct metabolic responses rather than completely different metabolic pathways.

The HAD and ND groups exhibited distinct metabolite–volatile correlation characteristics (Fig. 6C). The metabolic network analysis suggested that multiple metabolic modules, including lipid- and phenylpropanoid-related pathways, were involved in the differences between these treatments (Fig. S8C). Several metabolites associated with oxidative metabolism showed correlations with oxidation-derived volatile compounds. This pattern may be related to the prolonged oxygen exposure during natural drying, whereas HAD mainly induced temperature-dependent metabolic transformation.

A pronounced separation was observed between MD and VFD in metabolite–volatile correlation patterns (Fig. 6D). Metabolic network analysis indicated that valine, leucine and isoleucine degradation (ath00280) and flavonoid biosynthesis (ath00941) were among the pathways associated with metabolite differences (Fig. S8D). Flavonoid-related metabolites showed stronger associations with characteristic volatile compounds in VFD, whereas MD exhibited closer relationships with amino acid-related metabolites. These results suggest that microwave treatment was associated with greater metabolic differences, whereas VFD showed relatively higher abundance of several endogenous metabolites.

The ND and VFD groups showed highly divergent metabolite–volatile correlation patterns (Fig. 6E). Metabolic network analysis revealed that carbohydrate-related metabolism, including starch and sucrose metabolism (ath00500), was associated with differences between these treatments (Fig. S8E). Several carbohydrate-related metabolites showed correlations with volatile compounds, suggesting that prolonged environmental exposure during ND may alter carbohydrate metabolism, whereas VFD retained relatively stable endogenous metabolic profiles.

In contrast, ND and MD showed relatively similar metabolite–volatile correlation patterns (Fig. 6F). Consistent with the metabolic network analysis (Fig. S8F), no single dominant pathway was identified. Instead, limited metabolites associated with amino acid and flavonoid metabolism showed correlations with volatile compounds. These results suggest that ND and MD generated relatively comparable overall metabolic profiles compared with the larger differences observed between VFD and other drying treatments.

Overall, metabolite–volatile correlation analysis demonstrated that different drying treatments generated distinct associations between metabolic variation and volatile profiles. VFD was mainly associated with retention of several thermolabile metabolites and aroma-related compounds, whereas HAD and MD showed stronger associations with thermal transformation-related metabolites. ND showed metabolic characteristics associated with prolonged environmental exposure.

Similar trends have been reported in other chrysanthemum cultivars. Studies on Gongju and Hangbaiju showed that low-temperature drying generally favored retention of phenolic compounds and volatile precursors, whereas prolonged thermal or natural drying altered secondary metabolism and aroma profiles (Shi et al., 2017; Wang et al., 2018; Wang et al., 2019). The present results obtained from Chuju were generally consistent with these observations, while also suggesting cultivar-specific differences related to its characteristic aroma composition. However, because this study was based on correlation analysis and relative metabolite abundance, further targeted validation is required to confirm the direct biochemical relationships between specific metabolites and volatile compounds.

## 4 Conclusions

This multi-omics study systematically evaluated the effects of four drying strategies (ND, HAD, MD, and VFD) on aroma characteristics and metabolic profiles of Chuju. By integrating sensory evaluation, GC–IMS-based volatile fingerprinting, and untargeted metabolomics, this study revealed the associations between drying conditions, volatile variation, and metabolic changes, providing insights beyond conventional quality evaluation based only on individual physicochemical indicators. Different drying methods resulted in distinct volatile profiles and metabolite accumulation patterns, which were mainly associated with changes in amino acid-related metabolism, lipid-associated metabolism, and phenylpropanoid/flavonoid-related pathways.

Among the evaluated drying methods, VFD showed higher floral and green-grass aroma intensities and relatively higher levels of several aroma-associated volatile compounds and secondary metabolites. These characteristics were likely associated with the low-temperature and oxygen-limited conditions during freeze-drying, which may contribute to the preservation of thermolabile compounds. However, the longer processing duration, higher equipment requirements, and potentially higher energy consumption and economic costs may restrict its application in large-scale production. Therefore, VFD may be more suitable for high-value Chuju products where aroma preservation is prioritized.

HAD exhibited stronger sweet and honey-like aroma characteristics, which were associated with increased abundance of several thermal transformation-related volatile compounds and metabolites. However, prolonged heat exposure may also affect the retention of some thermolabile aroma components. MD provided rapid dehydration with intermediate aroma and metabolic characteristics between HAD and VFD, suggesting its potential as an alternative processing strategy. However, further studies considering energy consumption, processing cost, and scale-up performance are required before industrial application. ND maintained the traditional drying process but showed greater sample variation and weaker aroma characteristics, which may be related to prolonged environmental exposure during drying.

Overall, this study demonstrates that different drying methods were associated with distinct aroma and metabolic profiles in Chuju. The integration of volatile profiling and metabolomics provides a comprehensive framework for understanding drying-associated quality variation and supporting the selection of suitable processing strategies.

Several limitations should be acknowledged. First, GC–IMS analysis provided relative semi-quantitative volatile profiles, and further validation using authentic standards, quantitative calibration, and matrix-effect correction is required for accurate determination of aroma-active compounds. Second, although ND represents a traditional drying method, the prolonged drying duration may involve microbial succession, and the potential contribution of microbial communities to metabolite transformation and aroma evolution was not investigated. Future studies integrating microbiome analysis with metabolomics will help clarify the role of microorganisms during natural drying. Third, this study focused on the final dried products, and the dynamic evolution of metabolites and volatile compounds throughout the drying process remains unclear. Time-resolved multi-omics approaches are therefore needed to elucidate the progressive changes occurring during dehydration. Finally, this study mainly evaluated aroma characteristics and metabolic profiles, while other quality attributes, including color, appearance, and rehydration properties, were not investigated. Future studies integrating comprehensive quality evaluation and processing optimization will further improve the understanding and practical application of Chuju drying technologies.

## Declarations

Since the research involved tasting and food quality evaluation, the academic committee of the School of Biological and Food Engineering of Chuzhou University approved the study and exempted it from review. According to the FDA, CFR-Code of Federal Regulations Title 21-food quality and consumer acceptance studies are exempted from review (US Food and Drug Administration 2017).

## CRediT authorship contribution statement

**Yu-fang Luo:** Writing – original draft, Methodology, Investigation, Conceptualization. **Sam Al-Dalali:** Writing – review & editing. **Su-ting Gao:** Methodology, Investigation. **Qi Shen:** Methodology, Investigation. **Yuan Fu:** Investigation. **Yu-min Zhang:** Methodology, Investigation. **Wen Nie:** Writing – review & editing, Supervision, Conceptualization. **Ye-ye Du:** Writing – review & editing, Supervision, Conceptualization.

## Funding sources

This study was funded by the Key Project of Natural Science Research in Universities of Anhui Province (2024AH051431; 2024AH051416); Research Start-up Fund of Chuzhou University (2023qd33; 2023qd38; 2023qd32); Open Project of Anhui Engineering Research Center for Heat-Sensitive Materials Processing (2025RMZX03; 2025RMZX06; 2025RMZX05).

## Declaration of competing interest

The authors declare that they have no known competing financial interests or personal relationships that could have appeared to influence the work reported in this paper.

## Data Availability

I have shared the link to my data at the Attech File Step
