# Supplementary material for: Effects of different drying methods on the aroma and metabolic profiles of Chrysanthemum morifolium cv. Chuju
Source: Food Chem X. 2026 Oct 21;40:104634. doi: 10.1016/j.fochx.2026.104634 (PMC13717093; doi:10.1016/j.fochx.2026.104634)
Supplement: Supplementary file 1 [file mmc1.docx]

**Fig. S1.** Schematic diagram of the drying procedures applied for Chuju preparation. The diagram presents the main processing steps and key parameters, including drying temperature, microwave power, vacuum pressure, and processing duration for each drying method.

**Fig. S2.** Photographs of the sensory evaluation laboratory. (A) Overall view of the sensory evaluation room; (B) Individual sensory evaluation booth; (C) Laboratory environment and evaluation facilities.

**Fig. S3.** Determination of the optimal number of clusters for K-means analysis. (A) Elbow curve showing the within-cluster sum of squares (WSS) for different cluster numbers (k = 1–10). The inflection point occurred around k = 4. (B) Silhouette coefficient for different cluster numbers. The maximum silhouette coefficient was obtained at k = 3. Considering both clustering performance and biological interpretability, k = 3 was selected for subsequent K-means clustering analysis.

**Fig. S4.** Global metabolomic profiling of Chuju under four drying methods and multivariate analysis of the metabolomic dataset. Total ion current (TIC) chromatograms obtained in negative (A) and positive (B) ionization modes. (C) Overview of identified metabolites. (D) Proportions of metabolite superclasses. (E) Two-dimensional PCA score plot of Chuju samples subjected to four drying methods (HAD, ND, VFD, and MD; n = 3). (F) Three-dimensional PCA score plot based on PC1, PC2, and PC3. (G) One-dimensional distribution of PC1 scores. (H) One-dimensional distribution of PC2 scores. The solid, dashed, and dotted lines represent the mean, ±2 SD, and ±3 SD, respectively. (I) PCA score plot including QC samples.

**Fig. S5.** Permutation test validation of OPLS‑DA models for pairwise comparisons. (A) HAD vs. VFD, (B) HAD vs. MD, (C) HAD vs. ND, (D) MD vs. VFD, (E) ND vs. VFD, (F) ND vs. MD.

**Fig. S6.** K-means clustering analysis of differentially accumulated metabolites in Chuju under four drying methods. Differential metabolites were grouped into three clusters based on their standardized abundance patterns across HAD, ND, VFD, and MD treatments (k = 3). The optimal cluster number was determined using the Elbow Method and Silhouette Coefficient (Fig. S2). Each line represents an individual differential metabolite, and the black line indicates the average abundance trend within each cluster. The number of metabolites assigned to each cluster is indicated above each panel.

**Fig. S7.** Volcano plots of differentially accumulated metabolites between pairwise drying groups (VIP > 1, *P* < 0.05, |log₂FC| > 1). (A) HAD vs. VFD, (B) HAD vs. MD, (C) HAD vs. ND, (D) MD vs. VFD, (E) ND vs. VFD, (F) ND vs. MD. Red dots: up‑regulated metabolites; blue dots: down‑regulated metabolites; gray dots: no significant difference. Dot size represents the variable importance in projection (VIP) value.

**Fig.** **S8.** Metabolic‑regulatory network analysis of differential metabolites and pathways. (A) HAD vs. VFD, (B) HAD vs. MD, (C) HAD vs. ND, (D) MD vs. VFD, (E) ND vs. VFD, (F) ND vs. MD. Nodes represent metabolites, enzymes, and reactions; colors indicate module types.

**Table. S1.** Definition and reference standards of aroma descriptors for Chuju using quantitative descriptive analysis (QDA)

| Aroma characteristics | Definition | Reference | Score (0 = None, 9 = Very much) |
| --- | --- | --- | --- |
| Flowery | The unique fresh and elegant core floral fragrance of chrysanthemum varieties, with no off-odor | Fresh dried Chuju (no sulfur fumigation, no aging) |  |
| Sweet & Honey | A sweet and mellow aroma similar to honey or caramel, or a soft sweet fragrance of ripe berries | Acacia honey (mild flavor), ripe pear flesh (sweet type) |  |
| Green-Grass | The fresh, tender and refreshing aroma of fresh herbs, without any harsh or pungent greenness | Fresh and tender mugwort, freshly picked fresh leaves of Chuju chrysanthemum |  |
| Fruity | A delicate fruity aroma similar to apples and pears, without a strong fermented fruit flavor | Crisp and sweet green apples (peeled), fresh snow pears (flesh) |  |
| Woody | A slight woody, mellow and rich aroma from aging | Aged nanmu wood chips (light flavor), dried Chuju flowers naturally aged for 1 year |  |
| Burnt/Roasted | The aroma of roasted nuts or the burnt smell from overheating | Lightly roasted white sesame seeds (with a mild fragrance), slightly charred dried chrysanthemum flowers |  |

**Table. S1. (continued)**

| Aroma characteristics | Definition | Reference | Score (0 = None, 9 = Very much) |
| --- | --- | --- | --- |
| Stale & Musty | Stale, damp musty smell, earthy smell | Dried flowers of Chuju that have been damp and aged for more than 3 years, and dried herbs with a slight musty smell |  |

**Table. S2.** Response characteristics of PEN3 electronic nose sensors.

| No. | Sensors | Sensitivity target |
| --- | --- | --- |
| 1 | W1C | Aromatic compounds |
| 2 | W5S | Nitrogen oxides |
| 3 | W3C | Ammonia, aromatic compounds |
| 4 | W6S | Hydrides |
| 5 | W5C | Short-chain alkane, aromatic compounds |
| 6 | W1S | Methyl compounds |
| 7 | W1W | Sulfides, terpenes |
| 8 | W2S | Alcohols, aldehyde, ketone compounds |
| 9  10 | W2W | Aromatic,, organic, sulfides compounds |
|  | W3S | Long-chain alkanes |

**Table. S3.** Top 10 up- and down-regulated differential metabolites identified in pairwise comparisons among Chuju samples subjected to different drying methods.

| Comparison | Metabolite | KEGG Compound ID | Super Class | Class | Regulation |
| --- | --- | --- | --- | --- | --- |
| HAD vs VFD | Udp xylose | C00190 | Carbohydrates | Nucleosides | Down |
|  | Semilepidinoside B |  | Others | Others | Down |
|  | N-methyl-2-pyrrolidone |  | Alkaloids | Other alkaloids | Up |
|  | Jasmonic Acid | C08491 | Lipids | Eicosanoids | Up |
|  | Dihomo-gamma-linolenoylcholine [M] |  | Lipids | Fatty Acids and Conjugates | Down |
|  | Cer(d18:0/20:4) |  | Lipids | Ceramides | Up |
|  | Carboxystrictosidine |  | Others | Others | Up |
|  | Borneol, (-)- | C01411 | Terpenoids | Monoterpenoids | Up |
|  | Avenanthramide C |  | Others | Others | Down |
|  | 4-[2-(3,4-dihydroxyphenyl)ethenyl]-1,7-dihydroxy-2,3,5-trioxabicyclo[2.2.2]oct-7-en-6-one |  | Phenylpropanoids | Other Phenylpropanoids | Down |
|  | 3-Methoxytyrosine |  | Amino acids and derivatives | Amino acid derivatives | Down |

**Table. S3. (continued)**

| Comparison | Metabolite | KEGG Compound ID | Super Class | Class | Regulation |
| --- | --- | --- | --- | --- | --- |
|  | 3-Hydroxydecanedioylcarnitine |  | Lipids | Carnitines | Down |
|  | 2-(2,3-Dihydro-4-methoxyfuro(2,3-b)quinolin-2-yl)-1,2-propanediol |  | Alkaloids | Other alkaloids | Down |
|  | 2-(((2S,3R,4S,5S,6R)-3,4,5-Trihydroxy-6-(hydroxymethyl)tetrahydro-2H-pyran-2-yl)oxy)benzoic acid |  | Phenylpropanoids | Phenolic acids | Down |
|  | 12(S)-HHTrE | C20388 | Lipids | Fatty Acids and Conjugates | Down |
|  | 1-(2,4,5-Trimethoxyphenyl)-1,2-propanedione |  | Phenylpropanoids | Other Phenylpropanoids | Up |
|  | [2-Hydroxy-3-[3,4,5-trihydroxy-6-(hydroxymethyl)oxan-2-yl]oxypropyl] octadeca-9,12,15-trienoate |  | Lipids | Glycerolipids | Up |
|  | (S)-Abscisic acid |  | Terpenoids | Carotenoids | Up |

**Table. S3. (continued)**

| Comparison | Metabolite | KEGG Compound ID | Super Class | Class | Regulation |
| --- | --- | --- | --- | --- | --- |
|  | (2R,3S,4S,5R,6S)-2-(hydroxymethyl)-6-[1-methyl-1-[(1R)-4-methylcyclohex-3-en-1-yl]ethoxy]tetrahydropyran-3,4,5-triol |  | Terpenoids | Monoterpenoids | Up |
|  | (1S,2R,9R,11R,12R,15R)-11-hydroxy-2,6,9,15-tetramethyl-13-oxatetracyclo[10.2.1.02,10.05,9]pentadeca-4,6-diene-3,8,14-trione |  | Terpenoids | Sesquiterpenoids | Up |
| HAD vs MD | Uridine | C00299 | Carbohydrates | Nucleosides | Down |
|  | Thr-Leu |  | Amino acids and derivatives | Dipeptides | Down |
|  | Scutellarein | C10184 | Phenylpropanoids | Flavonoids | Down |
|  | Ovoic acid |  | Polyketides | Aromatic polyketides | Up |
|  | Okanin | C08724 | Phenylpropanoids | Flavonoids | Down |
|  | Neobignonoside |  | Phenylpropanoids | Flavonoids | Up |

**Table. S3. (continued)**

| Comparison | Metabolite | KEGG Compound ID | Super Class | Class | Regulation |
| --- | --- | --- | --- | --- | --- |
|  | N-Benzyloxycarbonylglycine | C03710 | Amino acids and derivatives | Amino acid derivatives | Up |
|  | Kiwiionoside |  | Terpenoids | Carotenoids | Up |
|  | Guanosine | C00387 | Carbohydrates | Nucleosides | Down |
|  | Broussoflavonol B |  | Phenylpropanoids | Flavonoids | Up |
|  | Angoroside A |  | Phenylpropanoids | Other Phenylpropanoids | Up |
|  | 7-(2-Hydroxypropan-2-yl)-1,4a-dimethyl-2,3,4,5,6,7,8,8a-octahydronaphthalen-1-ol |  | Terpenoids | Sesquiterpenoids | Down |
|  | 3,4',5,7-Tetrahydroxy-3'-methoxyflavylium |  | Phenylpropanoids | Anthocyanidins | Down |
|  | 3,4-Dicaffeoyl-1,5-quinolactone |  | Phenylpropanoids | Other Phenylpropanoids | Up |
|  | 2,6-Dihydroxybenzoic acid | C21298 | Phenylpropanoids | Phenolic acids | Down |
|  | 2,4,6-trihydroxybenzaldehyde |  | Polyketides | Aromatic polyketides | Down |
|  | 2-(3,4-dihydroxyphenyl)-5,7-dihydroxy-chroman-4-one |  | Phenylpropanoids | Flavonoids | Down |

**Table. S3. (continued)**

| Comparison | Metabolite | KEGG Compound ID | Super Class | Class | Regulation |
| --- | --- | --- | --- | --- | --- |
|  | 1-Hydroxy-3,5-dimethoxy-2,4-diprenylxanthone |  | Phenylpropanoids | Other Phenylpropanoids | Up |
|  | 1-(3-(3-methyl-2-butenyl)-2,4-(dihydroxy)phenyl)-3-(2,2-dimethyl-8-hydroxy-2H-benzopyran-6-yl)-2-propen-1-one |  | Phenylpropanoids | Flavonoids | Up |
|  | [3,4,5-Trihydroxy-6-[4-(3-oxobutyl)phenoxy]oxan-2-yl]methyl 3,4,5-trihydroxybenzoate |  | Phenylpropanoids | Phenolic acids | Up |
| HAD vs ND | Vinetorin |  | Polyketides | Other polyketides | Down |
|  | Udp xylose | C00190 | Carbohydrates | Nucleosides | Down |
|  | Semilepidinoside B |  | Others | Others | Down |
|  | Malonic acid | C00383 | Organic acids and derivatives | Carboxylic acids | Down |
|  | LPC(19:0) |  | Lipids | Glycerophospholipids | Up |

**Table. S3. (continued)**

| Comparison | Metabolite | KEGG Compound ID | Super Class | Class | Regulation |
| --- | --- | --- | --- | --- | --- |
|  | LPC(15:0) |  | Lipids | Glycerophospholipids | Up |
|  | Heterodendrin |  | Others | Others | Down |
|  | Germanasim E |  | Phenylpropanoids | Isoflavonoids | UP |
|  | Eugenol O-[3,4,5-Trihydroxybenzoyl-(->6)-b-glucopyranoside] |  | Phenylpropanoids | Other Phenylpropanoids | Up |
|  | Erysenegalensein J |  | Phenylpropanoids | Isoflavonoids | Up |
|  | Avenanthramide C |  | Other | Others | Down |
|  | 4,2',3',4',6'-Pentahydroxy-5'-neryldihydrochalcone |  | Phenylpropanoids | Flavonoids | Down |
|  | 3,4,5-Tris[3-(3,4-dihydroxyphenyl)prop-2-enoyloxy]-1-hydroxycyclohexane-1-carboxylic acid |  | Phenylpropanoids | Other Phenylpropanoids | Up |
|  | 3-Hydroxypyruvic acid | C00168 | Organic acids and derivatives | Hydroxy acids | Down |
|  | 2,6-Dihydroxybenzoic acid | C21298 | Phenylpropanoids | Phenolic acids | Down |

**Table. S3. (continued)**

| Comparison | Metabolite | KEGG Compound ID | Super Class | Class | Regulation |
| --- | --- | --- | --- | --- | --- |
|  | 2,4,6-trihydroxybenzaldehyde |  | Polyketides | Aromatic polyketides | Down |
|  | 1-(3-hydroxy-1b,2-dimethyl-2,3,4,5,5a,6-hexahydro-1aH-indeno[1,2-b]oxiren-6a-yl)ethanone |  | Alkaloids | Other alkaloids | Up |
|  | (Z)-3-(4-oxo-5-propan-2-ylidene-2-propylfuran-3-yl)prop-2-enoic acid |  | Polyketides | Other polyketides | Up |
|  | (2R)-8-ethyl-7-hydroxy-5-methoxy-2-methyl-chroman-4-one |  | Polyketides | Other polyketides | Up |
|  | (1S,4aR,5S,7aS)-1,4a,5,7a-Tetrahydro-5-[(4-hydroxybenzoyl)oxy]-7-(hydroxymethyl)cyclopenta[c]pyran-1-yl beta-D-glucopyranoside |  | Terpenoids | Monoterpenoids | Up |
| VFD vs MD | Tryptophyl-Alanine |  | Amino acids and derivatives | Dipeptides | Down |
|  | Proacacipetalin |  | Others | Others | Down |

**Table. S3. (continued)**

| Comparison | Metabolite | KEGG Compound ID | Super Class | Class | Regulation |
| --- | --- | --- | --- | --- | --- |
|  | N-methyl-2-pyrrolidone |  | Alkaloids | Other alkaloids | Up |
|  | N-Benzyloxycarbonylglycine | C03710 | Amino acids and derivatives | Amino acid derivatives | Down |
|  | Malic acid | C00149 | Organic acids and derivatives | Hydroxy acids | Up |
|  | Jasmonic Acid | C08491 | Lipids | Eicosanoids | Up |
|  | Isorhoifolin |  | Phenylpropanoids | Flavonoids | Up |
|  | Flavanomarein |  | Phenylpropanoids | Flavonoids | Down |
|  | Enicoflavine |  | Alkaloids | Other alkaloids | Down |
|  | Echitovenine |  | Alkaloids | Other alkaloids | Up |
|  | Cytidine | C00475 | Carbohydrates | Nucleosides | Up |
|  | Cer(d18:0/20:4) |  | Lipids | Ceramides | Up |
|  | Avenanthramide C |  | Others | Others | Down |
|  | 5'-Methylthioadenosine | C00170 | Carbohydrates | Nucleosides | Down |

**Table. S3. (continued)**

| Comparison | Metabolite | KEGG Compound ID | Super Class | Class | Regulation |
| --- | --- | --- | --- | --- | --- |
|  | 2-Aminoadipic acid | C00956 | Amino acids and derivatives | Amino acid derivatives | Down |
|  | 12,15-Dihydroxy-13-methylhexadecanoic acid |  | Lipids | Fatty Acids and Conjugates | Up |
|  | 1-[2,4-dihydroxy-6-[(2S,3R,4S,5S,6R)-3,4,5-trihydroxy-6-(hydroxymethyl)tetrahydropyran-2-yl]oxy-phenyl]ethanone |  | Polyketides | Aromatic polyketides | Down |
|  | (2R,3S,4S,5R,6S)-2-(hydroxymethyl)-6-[1-methyl-1-[(1R)-4-methylcyclohex-3-en-1-yl]ethoxy]tetrahydropyran-3,4,5-triol |  | Terpenoids | Monoterpenoids | Up |
|  | (1S,19R)-8,10-dioxa-4,17-diazaheptacyclo[15.4.3.01,18.04,19.05,13.07,11.014,19]tetracosa-5,7(11),12,22-tetraen-3-one |  | Alkaloids | Other alkaloids | Down |

**Table. S3. (continued)**

| Comparison | Metabolite | KEGG Compound ID | Super Class | Class | Regulation |
| --- | --- | --- | --- | --- | --- |
| ND vs VFD | Quercetin | C00389 | Phenylpropanoids | Flavonoids | Up |
|  | Nigrosporapyrone D |  | Polyketides | Macrolides | Down |
|  | myrsinoic acid B |  | Alkaloids | Other alkaloids | Up |
|  | LPC(18:0/0:0) |  | Lipids | Glycerophospholipids | Down |
|  | LPC(15:0) |  | Lipids | Glycerophospholipids | Down |
|  | Jasmonic Acid | C08491 | Lipids | Eicosanoids | Up |
|  | hexyl hexanoate |  | Lipids | Fatty Acids and Conjugates | Up |
|  | Dodecanoic acid | C02679 | Lipids | Fatty Acids and Conjugates | Up |
|  | Dihydroelymoclavine |  | Alkaloids | Other alkaloids | Up |
|  | Cytidine | C00475 | Carbohydrates | Nucleosides | Up |
|  | Crocin 5 |  | Lipids | Saccharolipids | Down |

**Table. S3. (continued)**

| Comparison | Metabolite | KEGG Compound ID | Super Class | Class | Regulation |
| --- | --- | --- | --- | --- | --- |
|  | 3,4,5-Tris[3-(3,4-dihydroxyphenyl)prop-2-enoyloxy]-1-hydroxycyclohexane-1-carboxylic acid |  | Phenylpropanoids | Other Phenylpropanoids | Down |
|  | 2-Hydroxyquinoline |  | Alkaloids | Other alkaloids | Up |
|  | 12,15-Dihydroxy-13-methylhexadecanoic acid |  | Lipids | Fatty Acids and Conjugates | Up |
|  | 1-palmitoyl-2-vernoloyl-phosphatidylcholine |  | Lipids | Glycerophospholipids | Down |
|  | 1-methyl-2,3,4,9-tetrahydro-1H-pyrido[3,4-b]indole-3-carboxylic acid |  | Alkaloids | Other alkaloids | Down |
|  | (Z)-3-(4-oxo-5-propan-2-ylidene-2-propylfuran-3-yl)prop-2-enoic acid |  | Polyketides | Other polyketides | Down |
|  | (2R)-8-ethyl-7-hydroxy-5-methoxy-2-methyl-chroman-4-one |  | Polyketides | Other polyketides | Down |

**Table. S3. (continued)**

| Comparison | Metabolite | KEGG Compound ID | Super Class | Class | Regulation |
| --- | --- | --- | --- | --- | --- |
|  | (2R,3S,4S,5R,6S)-2-(hydroxymethyl)-6-[1-methyl-1-[(1R)-4-methylcyclohex-3-en-1-yl]ethoxy]tetrahydropyran-3,4,5-triol |  | Terpenoids | Monoterpenoids | Up |
|  | (1R,4aS,10aR)-7-Isopropyl-1,4a-dimethyl-1,2,3,4,4a,9,10,10a-octahydrophenanthrene-1-carboxylic acid |  | Terpenoids | Diterpenoids | Down |
| ND vs MD | Synephrine | C04548 | Alkaloids | Other alkaloids | Up |
|  | Sinapic acid |  | Phenylpropanoids | Other Phenylpropanoids | Up |
|  | Proacacipetalin |  | Others | Others | Up |
|  | Phenylephrine | C07441 | Alkaloids | Other alkaloids | Up |
|  | methoxymaysin |  | Phenylpropanoids | Flavonoids | Down |
|  | Gluconic acid | C00257 | Carbohydrates | Saccharides and glycosides | Down |
|  | Eugenol O-[3,4,5-Trihydroxybenzoyl-(->6)-b-glucopyranoside] |  | Phenylpropanoids | Other Phenylpropanoids | Down |

**Table. S3. (continued)**

| Comparison | Metabolite | KEGG Compound ID | Super Class | Class | Regulation |
| --- | --- | --- | --- | --- | --- |
|  | Enicoflavine |  | Alkaloids | Other alkaloids | Up |
|  | Cyclohexylamine | C00571 | Others | Others | Up |
|  | Crocin 5 |  | Lipids | Saccharolipids | Down |
|  | Avenanthramide C |  | Others | Others | Up |
|  | Alanyltryptophan |  | Amino acids and derivatives | Dipeptides | Up |
|  | 5'-Methylthioadenosine | C00170 | Carbohydrates | Nucleosides | Up |
|  | 3,7-Dimethyluric acid | C16360 | Alkaloids | Purine alkaloids | Down |
|  | 3-[1-hydroxy-2-(methylamino)ethyl]phenol |  | Alkaloids | Other alkaloids | Up |
|  | 1-palmitoyl-2-vernoloyl-phosphatidylcholine |  | Lipids | Glycerophospholipids | Down |
|  | 1-(3-hydroxy-1b,2-dimethyl-2,3,4,5,5a,6-hexahydro-1aH-indeno[1,2-b]oxiren-6a-yl)ethanone |  | Alkaloids | Other alkaloids | Down |
|  | (Z)-3-(4-oxo-5-propan-2-ylidene-2-propylfuran-3-yl)prop-2-enoic acid |  | Polyketides | Other polyketides | Down |

**Table. S3. (continued)**

| Comparison | Metabolite | KEGG Compound ID | Super Class | Class | Regulation |
| --- | --- | --- | --- | --- | --- |
|  | (2R)-8-ethyl-7-hydroxy-5-methoxy-2-methyl-chroman-4-one |  | Polyketides | Other polyketides | Down |
|  | (2E,6E,10E)-12-acetyloxy-6,10-dimethyl-2-(4-methylpent-3-enyl)dodeca-2,6,10-trienoic acid |  | Terpenoids | Diterpenoids | Down |

Differential metabolites were screened using VIP > 1, *P* < 0.05, and FC ≥ 2 or FC ≤ 0.5. For each pairwise comparison, the top 10 up-regulated metabolites were selected based on the largest FC values, whereas the top 10 down-regulated metabolites were selected based on the smallest FC values. “Up-regulated” indicates FC ≥ 2, whereas “Down-regulated” indicates FC ≤ 0.5.

**Table. S4.** KEGG pathway annotation information of differential metabolites identified in this study.

| KEGG Pathway ID | Description | Class |
| --- | --- | --- |
| ath01100 | Metabolic pathways - Arabidopsis thaliana (thale cress) | Global and overview maps |
| ath01110 | Biosynthesis of secondary metabolites - Arabidopsis thaliana (thale cress) | Global and overview maps |
| ath01230 | Biosynthesis of amino acids - Arabidopsis thaliana (thale cress) | Global and overview maps |
| ath01240 | Biosynthesis of cofactors - Arabidopsis thaliana (thale cress) | Global and overview maps |
| ath01210 | 2-Oxocarboxylic acid metabolism - Arabidopsis thaliana (thale cress) | Global and overview maps |
| ath01232 | Nucleotide metabolism - Arabidopsis thaliana (thale cress) | Global and overview maps |
| ath00230 | Purine metabolism - Arabidopsis thaliana (thale cress) | Nucleotide metabolism |
| ath01200 | Carbon metabolism - Arabidopsis thaliana (thale cress) | Global and overview maps |
| ath00630 | Glyoxylate and dicarboxylate metabolism - Arabidopsis thaliana (thale cress) | Carbohydrate metabolism |
| ath00240 | Pyrimidine metabolism - Arabidopsis thaliana (thale cress) | Nucleotide metabolism |
| ath00290 | Valine, leucine and isoleucine biosynthesis - Arabidopsis thaliana (thale cress) | Amino acid metabolism |
| ath00470 | D-Amino acid metabolism - Arabidopsis thaliana (thale cress) | Metabolism of other amino acids |
| ath00260 | Glycine, serine and threonine metabolism - Arabidopsis thaliana (thale cress) | Amino acid metabolism |
| ath00330 | Arginine and proline metabolism - Arabidopsis thaliana (thale cress) | Amino acid metabolism |

**Table. S4.** **(continued)**

| KEGG Pathway ID | Description | Class |
| --- | --- | --- |
| ath00960 | Tropane, piperidine and pyridine alkaloid biosynthesis - Arabidopsis thaliana (thale cress) | Biosynthesis of other secondary metabolites |
| ath00640 | Propanoate metabolism - Arabidopsis thaliana (thale cress) | Carbohydrate metabolism |
| ath00944 | Flavone and flavonol biosynthesis - Arabidopsis thaliana (thale cress) | Biosynthesis of other secondary metabolites |
| ath00966 | Glucosinolate biosynthesis - Arabidopsis thaliana (thale cress) | Biosynthesis of other secondary metabolites |
| ath00999 | Biosynthesis of various plant secondary metabolites - Arabidopsis thaliana (thale cress) | Biosynthesis of other secondary metabolites |
| ath00061 | Fatty acid biosynthesis - Arabidopsis thaliana (thale cress) | Lipid metabolism |
| ath01040 | Biosynthesis of unsaturated fatty acids - Arabidopsis thaliana (thale cress) | Lipid metabolism |
| ath00310 | Lysine degradation - Arabidopsis thaliana (thale cress) | Amino acid metabolism |
| ath00460 | Cyanoamino acid metabolism - Arabidopsis thaliana (thale cress) | Metabolism of other amino acids |
| ath00940 | Phenylpropanoid biosynthesis - Arabidopsis thaliana (thale cress) | Biosynthesis of other secondary metabolites |
| ath00232 | Caffeine metabolism - Arabidopsis thaliana (thale cress) | Biosynthesis of other secondary metabolites |
| ath00592 | alpha-Linolenic acid metabolism - Arabidopsis thaliana (thale cress) | Lipid metabolism |

**Table. S4.** **(continued)**

| KEGG Pathway ID | Description | Class |
| --- | --- | --- |
| ath00280 | Valine, leucine and isoleucine degradation - Arabidopsis thaliana (thale cress) | Amino acid metabolism |
| ath00350 | Tyrosine metabolism - Arabidopsis thaliana (thale cress) | Amino acid metabolism |
| ath00360 | Phenylalanine metabolism - Arabidopsis thaliana (thale cress) | Amino acid metabolism |
| ath00760 | Nicotinate and nicotinamide metabolism - Arabidopsis thaliana (thale cress) | Metabolism of cofactors and vitamins |
| ath00770 | Pantothenate and CoA biosynthesis - Arabidopsis thaliana (thale cress) | Metabolism of cofactors and vitamins |
| ath00941 | Flavonoid biosynthesis - Arabidopsis thaliana (thale cress) | Biosynthesis of other secondary metabolites |
| ath00620 | Pyruvate metabolism - Arabidopsis thaliana (thale cress) | Carbohydrate metabolism |
| ath00591 | Linoleic acid metabolism - Arabidopsis thaliana (thale cress) | Lipid metabolism |
| ath00250 | Alanine, aspartate and glutamate metabolism - Arabidopsis thaliana (thale cress) | Amino acid metabolism |
| ath00270 | Cysteine and methionine metabolism - Arabidopsis thaliana (thale cress) | Amino acid metabolism |
| ath00380 | Tryptophan metabolism - Arabidopsis thaliana (thale cress) | Amino acid metabolism |
| ath00410 | beta-Alanine metabolism - Arabidopsis thaliana (thale cress) | Metabolism of other amino acids |
| ath01250 | Biosynthesis of nucleotide sugars - Arabidopsis thaliana (thale cress) | Global and overview maps |
| ath00020 | Citrate cycle (TCA cycle) - Arabidopsis thaliana (thale cress) | Carbohydrate metabolism |

**Table. S4.** **(continued)**

| KEGG Pathway ID | Description | Class |
| --- | --- | --- |
| ath00500 | Starch and sucrose metabolism - Arabidopsis thaliana (thale cress) | Carbohydrate metabolism |
| ath00660 | C5-Branched dibasic acid metabolism - Arabidopsis thaliana (thale cress) | Carbohydrate metabolism |
| ath00073 | Cutin, suberine and wax biosynthesis - Arabidopsis thaliana (thale cress) | Lipid metabolism |
| ath00300 | Lysine biosynthesis - Arabidopsis thaliana (thale cress) | Amino acid metabolism |
| ath00400 | Phenylalanine, tyrosine and tryptophan biosynthesis - Arabidopsis thaliana (thale cress) | Amino acid metabolism |
| ath00480 | Glutathione metabolism - Arabidopsis thaliana (thale cress) | Metabolism of other amino acids |
| ath00520 | Amino sugar and nucleotide sugar metabolism - Arabidopsis thaliana (thale cress) | Carbohydrate metabolism |
| ath00785 | Lipoic acid metabolism - Arabidopsis thaliana (thale cress) | Metabolism of cofactors and vitamins |
| ath00908 | Zeatin biosynthesis - Arabidopsis thaliana (thale cress) | Metabolism of terpenoids and polyketides |
| ath00942 | Anthocyanin biosynthesis - Arabidopsis thaliana (thale cress) | Biosynthesis of other secondary metabolites |
| ath00261 | Monobactam biosynthesis - Arabidopsis thaliana (thale cress) | Biosynthesis of other secondary metabolites |
| ath01212 | Fatty acid metabolism - Arabidopsis thaliana (thale cress) | Global and overview maps |
| ath00650 | Butanoate metabolism - Arabidopsis thaliana (thale cress) | Carbohydrate metabolism |

**Table. S4.** **(continued)**

| KEGG Pathway ID | Description | Class |
| --- | --- | --- |
| ath00710 | Carbon fixation by Calvin cycle - Arabidopsis thaliana (thale cress) | Energy metabolism |
| ath00600 | Sphingolipid metabolism - Arabidopsis thaliana (thale cress) | Lipid metabolism |
| ath00590 | Arachidonic acid metabolism - Arabidopsis thaliana (thale cress) | Lipid metabolism |
| ath00220 | Arginine biosynthesis - Arabidopsis thaliana (thale cress) | Amino acid metabolism |
| ath00730 | Thiamine metabolism - Arabidopsis thaliana (thale cress) | Metabolism of cofactors and vitamins |
| ath00740 | Riboflavin metabolism - Arabidopsis thaliana (thale cress) | Metabolism of cofactors and vitamins |
| ath00780 | Biotin metabolism - Arabidopsis thaliana (thale cress) | Metabolism of cofactors and vitamins |
| ath00670 | One carbon pool by folate - Arabidopsis thaliana (thale cress) | Metabolism of cofactors and vitamins |
| ath00860 | Porphyrin metabolism - Arabidopsis thaliana (thale cress) | Metabolism of cofactors and vitamins |
| ath00130 | Ubiquinone and other terpenoid-quinone biosynthesis - Arabidopsis thaliana (thale cress) | Metabolism of cofactors and vitamins |
| ath00030 | Pentose phosphate pathway - Arabidopsis thaliana (thale cress) | Carbohydrate metabolism |
| ath00051 | Fructose and mannose metabolism - Arabidopsis thaliana (thale cress) | Carbohydrate metabolism |
| ath00052 | Galactose metabolism - Arabidopsis thaliana (thale cress) | Carbohydrate metabolism |

**Table. S4.** **(continued)**

| KEGG Pathway ID | Description | Class |
| --- | --- | --- |
| ath00053 | Ascorbate and aldarate metabolism - Arabidopsis thaliana (thale cress) | Carbohydrate metabolism |
| ath00190 | Oxidative phosphorylation - Arabidopsis thaliana (thale cress) | Energy metabolism |
| ath00910 | Nitrogen metabolism - Arabidopsis thaliana (thale cress) | Energy metabolism |
| ath00920 | Sulfur metabolism - Arabidopsis thaliana (thale cress) | Energy metabolism |
| ath00062 | Fatty acid elongation - Arabidopsis thaliana (thale cress) | Lipid metabolism |
| ath00071 | Fatty acid degradation - Arabidopsis thaliana (thale cress) | Lipid metabolism |
| ath00100 | Steroid biosynthesis - Arabidopsis thaliana (thale cress) | Lipid metabolism |
| ath00561 | Glycerolipid metabolism - Arabidopsis thaliana (thale cress) | Lipid metabolism |
| ath00564 | Glycerophospholipid metabolism - Arabidopsis thaliana (thale cress) | Lipid metabolism |
| ath00565 | Ether lipid metabolism - Arabidopsis thaliana (thale cress) | Lipid metabolism |
| ath00430 | Taurine and hypotaurine metabolism - Arabidopsis thaliana (thale cress) | Metabolism of other amino acids |
| ath00440 | Phosphonate and phosphinate metabolism - Arabidopsis thaliana (thale cress) | Metabolism of other amino acids |
| ath00450 | Selenocompound metabolism - Arabidopsis thaliana (thale cress) | Metabolism of other amino acids |
| ath00541 | Biosynthesis of various nucleotide sugars - Arabidopsis thaliana (thale cress) | Glycan biosynthesis and metabolism |

**Table. S4.** **(continued)**

| KEGG Pathway ID | Description | Class |
| --- | --- | --- |
| ath00563 | Glycosylphosphatidylinositol (GPI)-anchor biosynthesis - Arabidopsis thaliana (thale cress) | Glycan biosynthesis and metabolism |
| ath00750 | Vitamin B6 metabolism - Arabidopsis thaliana (thale cress) | Metabolism of cofactors and vitamins |
| ath00790 | Folate biosynthesis - Arabidopsis thaliana (thale cress) | Metabolism of cofactors and vitamins |
| ath00902 | Monoterpenoid biosynthesis - Arabidopsis thaliana (thale cress) | Metabolism of terpenoids and polyketides |
| ath00906 | Carotenoid biosynthesis - Arabidopsis thaliana (thale cress) | Metabolism of terpenoids and polyketides |
| ath00950 | Isoquinoline alkaloid biosynthesis - Arabidopsis thaliana (thale cress) | Biosynthesis of other secondary metabolites |
| ath00965 | Betalain biosynthesis - Arabidopsis thaliana (thale cress) | Biosynthesis of other secondary metabolites |
| Pathway | Description | Class |
| ath00040 | Pentose and glucuronate interconversions - Arabidopsis thaliana (thale cress) | Carbohydrate metabolism |
| ath00195 | Photosynthesis - Arabidopsis thaliana (thale cress) | Energy metabolism |
| ath00945 | Stilbenoid, diarylheptanoid and gingerol biosynthesis - Arabidopsis thaliana (thale cress) | Biosynthesis of other secondary metabolites |
| ath00901 | Indole alkaloid biosynthesis - Arabidopsis thaliana (thale cress) | Biosynthesis of other secondary metabolites |

**Table. S4.** **(continued)**

| KEGG Pathway ID | Description | Class |
| --- | --- | --- |
| ath00562 | Inositol phosphate metabolism - Arabidopsis thaliana (thale cress) | Carbohydrate metabolism |
| ath00340 | Histidine metabolism - Arabidopsis thaliana (thale cress) | Amino acid metabolism |
